# Spatiotemporal Trends in Self-Reported Mask-Wearing Behavior in the United States: Analysis of a Large Cross-sectional Survey

**DOI:** 10.2196/42128

**Published:** 2023-03-06

**Authors:** Juliana C Taube, Zachary Susswein, Shweta Bansal

**Affiliations:** 1 Department of Biology Georgetown University Washington, DC United States

**Keywords:** COVID-19, face mask, nonpharmaceutical interventions, spatiotemporal, United States, survey bias, survey, US, behavior, effectiveness, disease, decision-making, community, surveillance

## Abstract

**Background:**

Face mask wearing has been identified as an effective strategy to prevent the transmission of SARS-CoV-2, yet mask mandates were never imposed nationally in the United States. This decision resulted in a patchwork of local policies and varying compliance, potentially generating heterogeneities in the local trajectories of COVID-19 in the United States. Although numerous studies have investigated the patterns and predictors of masking behavior nationally, most suffer from survey biases and none have been able to characterize mask wearing at fine spatial scales across the United States through different phases of the pandemic.

**Objective:**

Urgently needed is a debiased spatiotemporal characterization of mask-wearing behavior in the United States. This information is critical to further assess the effectiveness of masking, evaluate the drivers of transmission at different time points during the pandemic, and guide future public health decisions through, for example, forecasting disease surges.

**Methods:**

We analyzed spatiotemporal masking patterns in over 8 million behavioral survey responses from across the United States, starting in September 2020 through May 2021. We adjusted for sample size and representation using binomial regression models and survey raking, respectively, to produce county-level monthly estimates of masking behavior. We additionally debiased self-reported masking estimates using bias measures derived by comparing vaccination data from the same survey to official records at the county level. Lastly, we evaluated whether individuals’ perceptions of their social environment can serve as a less biased form of behavioral surveillance than self-reported data.

**Results:**

We found that county-level masking behavior was spatially heterogeneous along an urban-rural gradient, with mask wearing peaking in winter 2021 and declining sharply through May 2021. Our results identified regions where targeted public health efforts could have been most effective and suggest that individuals’ frequency of mask wearing may be influenced by national guidance and disease prevalence. We validated our bias correction approach by comparing debiased self-reported mask-wearing estimates with community-reported estimates, after addressing issues of a small sample size and representation. Self-reported behavior estimates were especially prone to social desirability and nonresponse biases, and our findings demonstrated that these biases can be reduced if individuals are asked to report on community rather than self behaviors.

**Conclusions:**

Our work highlights the importance of characterizing public health behaviors at fine spatiotemporal scales to capture heterogeneities that may drive outbreak trajectories. Our findings also emphasize the need for a standardized approach to incorporating behavioral big data into public health response efforts. Even large surveys are prone to bias; thus, we advocate for a social sensing approach to behavioral surveillance to enable more accurate estimates of health behaviors. Finally, we invite the public health and behavioral research communities to use our publicly available estimates to consider how bias-corrected behavioral estimates may improve our understanding of protective behaviors during crises and their impact on disease dynamics.

## Introduction

Human behavior plays a key role in infectious disease transmission [1,2]. Individuals’ decisions to get vaccinated, reduce their contacts, or wear a face mask, for example, can have a tremendous impact on disease dynamics [3-5]. The COVID-19 pandemic has highlighted that we are grossly limited in our ability to accurately measure and predict human behavior in the face of a novel pathogen. Yet, knowledge of how human behaviors vary over time and space is critical to assess the effectiveness of mitigation strategies, to forecast disease surges, and to parameterize coupled disease-behavior models [6]. In particular, there is a paucity of data on how the frequency of face mask wearing varies across the United States over different phases of the pandemic. This lack of fine-scale spatiotemporal data has forced public health organizations to adopt an inefficient one-size-fits-all approach to encourage masking nationwide, rather than directing resources and messaging to areas with the lowest uptake. Here, we identify the spatiotemporal trends in self-reported data on mask-wearing behavior across the United States from a large survey distributed from September 2020 to May 2021.

Mask wearing has been identified as an effective strategy to reduce the transmission of SARS-CoV-2. At the individual level, masks decrease both the amount of viral particles dispersed by an infectious wearer and the amount of those inhaled by an uninfected wearer [7]. Modeling studies at the population level (eg, [4,8,9]) have suggested that mask wearing can limit SARS-CoV-2 transmission and COVID-19 deaths, including under scenarios where masks are not worn universally or are not completely effective at blocking transmission. Randomized controlled trials (eg, [10]) have also demonstrated that mask wearing is an effective community-level intervention against COVID-19. Despite limited information at the time, the Centers for Disease Control and Prevention (CDC) initially recommended mask wearing on April 3, 2020 [11]. Lack of a national mandate, though, resulted in a heterogeneous landscape of mask policies across states, counties, towns, and even individual businesses in the United States [12,13]. Compounded with this spatial heterogeneity in mandates is additional heterogeneity in compliance, documented by localized observational studies (eg, [14]). A collection of systematic, accurate data on mask-wearing levels across the United States is therefore essential to informing our understanding of the role of mask wearing in the US COVID-19 pandemic trajectory.

To address this gap, researchers and organizations have implemented extensive surveys on human behavior, including mask wearing (eg, [15-17]). These surveys hold exciting promise, yet they have contributed relatively little to our understanding of human behavior, due to significant sampling limitations. Larger surveys with sufficient power to detect trends at local geographic scales are often not designed to capture a representative sample of the population. Demographic biases arising from a nonrepresentative sample can be addressed with standard statistical tools, such as survey weights, but other forms of bias, particularly nonresponse and social desirability biases, are more challenging to correct. Surveys about salient public health issues are especially likely to suffer from response bias; COVID-19–cautious individuals may be overrepresented in a survey about COVID-19 behavior. However, without estimates of the proportion of individuals in a given region who are COVID-19 cautious, there is no way to use survey weights on this demographic. Likewise, respondents may be influenced by social desirability bias when self-reporting COVID-19–preventive behaviors, such as vaccination, social distancing, or mask wearing, so that they respond in a manner deemed favorable by society despite being inaccurate [18]. Without observational or ground-truth data to validate survey responses, quantifying this social desirability bias is difficult. Furthermore, it is critical that ground-truth data to correct biases in health behavior be used at a fine spatial and temporal scale to avoid further exacerbation of biases (eg, [19]).

The value of surveys on public health behaviors can be further restricted when data collection is at the national or state level. Coarse-grained spatiotemporal information about human behavior is of limited utility, providing only sparse insight into local trends. Collecting responses at the national or state level ignores spatial heterogeneity at these finer scales, preventing the identification of these local effects that can drive disease dynamics. Spatial heterogeneity in not only drivers of disease transmission, such as human behavior, but also disease prevalence has been well documented across pathogens (eg, [20-22]). For example, differences in connectivity between counties or states can affect the timing and geographic scale of disease spread, while national scale mobility data elide these key patterns [23,24]. Likewise, aggregation of vaccination data to the state level can hide spatial clustering of unvaccinated individuals, which undermines herd immunity and could drive sustained measles outbreaks in the United States [25,26]. Despite the importance of detailed local estimates on the drivers of disease incidence, few studies have analyzed human behavior during the COVID-19 pandemic nationally at these fine spatial scales. Furthermore, most surveys are not conducted for long enough to capture human behavior changes over time, leaving scant opportunity to assess the effects of changing public health messaging/guidance or disease prevalence on human behavior.

Here, we systematically characterize mask wearing across the United States at a fine spatiotemporal scale for 9 months using a national survey and account for the bias in this survey. By comparing survey demographics and vaccination statuses with accurate ground-truth data, we estimate and account for survey and response biases in our analysis of masking behavior. With these bias-corrected estimates, we characterize the spatiotemporal heterogeneity in masking behavior at the county-month level across the United States. Finally, we examine the differences between self-reported and community-reported estimates of masking using an additional survey question, seeking to understand whether these 2 measures are good predictors of one another. Our results are the most precise estimates of masking in the United States during the COVID-19 pandemic, providing insight into the local variation in behavior in response to public health messaging and changes in COVID-19 incidence.

## Methods

### Study Design

In this study, we sought to characterize the spatiotemporal heterogeneity in self-reported masking behavior in the United States from the fall of 2020 to the spring of 2021. Due to the small sample size in some counties, we used Bayesian binomial regression models to estimate mask-wearing proportions each month. Recognizing that surveys are subject to several types of bias, we used raking and resampling of responses to correct for unrepresentative samples and self-reported vaccination status compared to ground-truth vaccination data to quantify nonresponse and social desirability biases. With these estimates, we were able to identify spatiotemporal trends in bias-corrected masking behavior and compare these values to reported community levels of masking in a different survey question.

### Survey Data and Processing

We analyzed self-reported mask-wearing survey responses for all 50 US states and the District of Columbia using data from the US COVID-19 Trends and Impact Survey (CTIS) [27]. The CTIS was created by the Delphi Research Group at Carnegie Mellon University and distributed through a partnership with Facebook. Beginning in September 2020, a random state-stratified sample of active Facebook users was invited daily to take the survey about COVID-19 and report how often they wore a mask in the past 5-7 days (the number of days changed from 5 to 7 on February 8, 2021). The answer options were (1) “All of the time,” (2) “Most of the time,” (3) “Some of the time,” (4) “A little of the time,” (5) “None of the time,” and (6) “I have not been in public in the last 5-7 days” (Multimedia Appendix 1, Figure S13). To dichotomize these responses for an analysis of the proportion of respondents wearing masks, we dropped respondents who had not been in public recently or did not respond to the masking question, and considered responses of “all of the time” and “most of the time” as masking, and all other responses as not masking. This cut-off is reasonable, considering the raw proportions of responses in each category for September through May (Multimedia Appendix 1, Figure S14). Due to sample size constraints, we aggregated these responses to the county-month scale. We ignored potential heterogeneity at smaller temporal (weekly) and spatial (zip code) scales due to the limited sample size.

By dichotomizing masking responses, we also lost information about the frequency with which people mask, though we expect the effect of this choice to be minimal (see Multimedia Appendix 1 for details).

### Bayesian Binomial Regression Model

Due to the small sample sizes in some US counties, we used Bayesian binomial regression models to develop reliable estimates of the proportion of individuals masking in a given county-month. Population density was used as a fixed effect; masking behavior has previously been linked to population density, and this variable was easily available at the county scale [14,28]. We fit separate models for each month, allowing for a temporal trend without explicitly modeling it by specifying a parametric form. We defined M_i_ as the number of respondents masking in county *i* (eg, respondents who masked most or all of the time in the past 5-7 days), N_i_ as the total number of respondents in county *i* (M_i_ ≤ N_i_), and *p*_i_ as the county-level probability of a response consistent with masking. We used the following model to estimate 
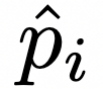
 and 
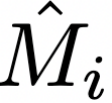
:



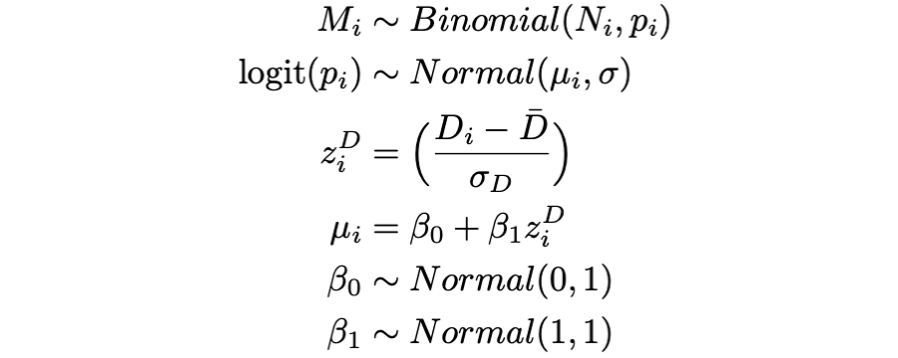



where D_i_ = log_10_(population density_i_) for county *i*. We ran the model using *brms* [29] with the *cmdstanR* [30] backend. We ran the sampler with 4 chains for 3000 iterations per chain. Sampler diagnostics indicated efficient exploration and that the model had converged, n_eff_ < 0.25, n_eff_ per iteration ≥ 0.25, 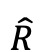 ≤ 1.01, E-BFMI > 0.25, and no transitions hit the maximum tree depth. All Pareto-smoothed importance sampling leave-one-out (PSIS-LOO) k statistic values were below 0.71, indicating that the model was robust to the influence of individual observations, and the distribution of Pareto k statistics was uniform, indicating that the model captured essential features of the data [31]. Posterior predictive checks indicated a good model fit (Multimedia Appendix 1, Figure S15) as did the plots of observed versus predicted and residual values (Multimedia Appendix 1, Figures S16 and S17). We note that our binomial regression approach compensated for the small sample sizes in some county-months, but the resulting estimates depend on the validity of our model structure.

We also explored more complex model specifications that included state- or county-level random effects. However, both models suffered from a lack of convergence or overfitting and produced functionally similar results. Thus, we opted for the more parsimonious model presented earlier for our main findings; details of these additional models can be found in Multimedia Appendix 1 (Figures S20 and S21).

### Survey Raking and Resampling

We were unable to use the provided weights for responses to the CTIS, due to spatial and temporal mismatch with the scales of our data analysis. Thus, we calculated county-month weights for each observation using the anesrake package [32] and the US Census American Community Survey’s 2018 5-year data on county age, sex, and education distributions. Age, sex, and education distributions were based on each county’s population over the age of 18 years to match the survey sample. We did not use race or ethnicity data in the raking scheme, as their inclusion substantially reduced algorithm convergence, but note that race/ethnicity was moderately correlated with education (Cramer's V>0.10). We then resampled from these responses using the calculated weights to estimate a raked masking proportion, which was fed into the binomial models, as described before. We excluded observations with missing age, sex, or education responses from the raking process and assigned equal weights to observations from county-months that did not converge (additional details in Multimedia Appendix 1).

### Estimation of CTIS Masking Bias

Given the likelihood of sampling, nonresponse, and social desirability biases, we generated bias-corrected estimates of masking in the United States. In the absence of ground-truth masking data with which to calibrate these CTIS responses, we turned to a different survey question for which ground-truth data were available.

Beginning in late December 2020, the CTIS asked respondents whether they had received a COVID-19 vaccine. The response options were (1) “Yes, “(2) “No,” and (3) “I don’t know.” Meanwhile, ground-truth vaccination data were collected by combining state-reported and CDC data to estimate the percentage of people vaccinated in each county in the United States [33,34]. A comparison of CTIS responses and ground-truth vaccination data revealed that the estimates of COVID-19 vaccination based on CTIS responses were much higher than true vaccination rates at the US county scale (Multimedia Appendix 1, Figures S18 and S19, [19,35]). Assuming that masking survey responses suffer from the same bias issues (in magnitude and direction) as vaccination responses, this result would suggest that CTIS responses also substantially overestimate masking behavior. Thus, we approximated survey bias by comparing the CTIS vaccination responses to the ground-truth vaccination data at the county level and incorporating this bias into the model of CTIS masking behavior.

Like the masking data, the CTIS vaccination response data suffers from small and unrepresentative samples in some counties. Thus, we resampled the responses from April and May 2021 according to the survey weights we generated before and then used a (frequentist) binomial generalized linear mixed-effects model to estimate *p*_i_, the proportion of respondents who were vaccinated (assumed to be partial vaccination, with 1 of a 1-dose or 2-dose vaccine) at the county level each week (details in Multimedia Appendix 1).

Given these modeled CTIS county-level vaccination proportions, we compared them with the true vaccination data to calculate the expected bias in reported survey responses relative to ground-truth data in county *i*:

bias_i_ = logit(CTIS estimated vaccination proportion_i_) – logit(true vaccination proportion_i_)

To increase the stability of our bias estimates, we used a linear mixed-effects model. This mixed-effects model used random intercepts, which penalizes extreme coefficient estimates to the overall mean, and assumed that the residual error in the estimates was normally distributed. This model generated a penalized estimate of survey bias for each county from the difference in modeled reported vaccination and ground-truth vaccination:



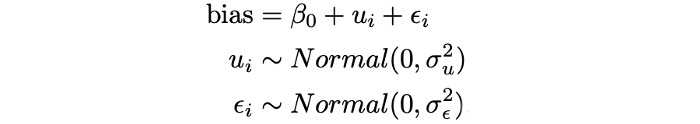



This model was implemented using *lmer* in the *lme4* package [36]. If there were no responses in county *i* or a bias estimate could not be calculated, bias estimates for this county were imputed by taking the mean of surrounding county estimates.

We then incorporated these estimates into a Bayesian binomial regression model with an offset for bias to estimate the bias-corrected probability of reporting masking in county *i*. We defined M_i_ as the number of respondents masking in county *i* out of N_i_ total respondents and *p*_i_ as the county-level probability of a response consistent with masking. We used the following model to estimate 
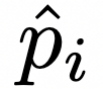
 and 
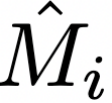
:



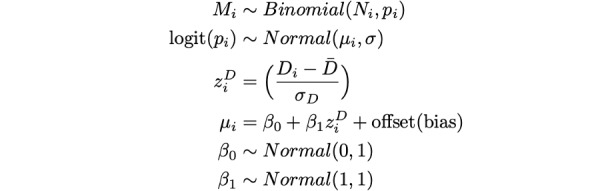



where D_i_ = log_10_(population density_i_) for county *i*. The bias-corrected proportion of individuals masking, c_i_, was calculated as



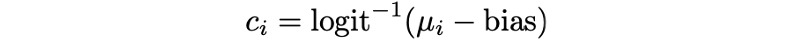



We ran the model using *brms* [29] with the *cmdstanR* [30] backend. We ran the sampler with 4 chains for 3000 iterations per chain. Sampler diagnostics indicated efficient exploration and that the model had converged: n_eff_>1000, n_eff_ per iteration≥0.3, and 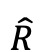 ≤ 1.01.

### Community-Reported Masking

Beginning November 24, 2020, the CTIS asked a question about masking in one’s community: “In the past 7 days, when out in public places where social distancing is not possible, about how many people would you estimate wore masks?” The answer options were (1) “All of the people,” (2) “Most of the people,” (3) “Some of the people,” (4) “A few of the people,” (5) “None of the people,” and (6) “I have not been out in public places in the past 7 days.” We dichotomized these responses and aggregated them to the county-month the same way as the self-reported CTIS masking responses for December 2020 through May 2021. We then modeled these community masking estimates the same way we modeled the CTIS masking data using Bayesian binomial regression and resampling weighted by survey weights but without a bias offset.

### Spatiotemporal Analysis

All analyses were completed in R version 4.1.3 (R Core Team and the R Foundation for Statistical Computing), and maps were produced using *choroplethr* [37]. Urban-rural classes were from the National Center for Health Statistics (NCHS) Urban-Rural Classification Scheme [38].

### Ethical Considerations

This study was reviewed by the Institutional Review Board of Georgetown University and was determined not to be human subject research.

## Results

To characterize the trends in the masking behavior in the United States during the COVID-19 pandemic, we used data from the CTIS conducted via Facebook from September 2020 through May 2021. Respondents self-reported how often they had worn a mask while in public in the past week (8,338,877 valid responses). We transformed these responses into a binary variable of masking or not masking and aggregated the responses to the county-month level to analyze spatiotemporal trends. To validate this data source, we analyzed a separate data set from Outbreaks Near Me and found consistent spatiotemporal patterns (Multimedia Appendix 1, Figures S1-S3), though both data sources suffer from issues of bias and small sample size. We addressed these issues in the CTIS data using binomial regression models to inform estimates of masking in counties with a small sample size, and raking and sample rebalancing on age, sex, and education to adjust for unrepresentative samples. Recognizing that CTIS responses to a question about vaccination overestimated the true vaccination rates, we quantified this bias for each county and used it to correct the estimates of masking behavior, assuming that vaccination and masking behavior responses were equally as biased. (Vaccination and mask wearing are both prosocial public health behaviors, which are socially desirable to report and are likely correlated [39-43].) We analyzed the overall spatial and temporal trends as well as fine-scale heterogeneity in the bias-corrected masking behavior estimates. Finally, we validated the bias-corrected CTIS values by comparing them to the respondents’ estimates of the proportion of people masking in their community.

### Spatially Heterogeneous Effects of the Binomial Regression Model, Survey Raking, and Debiasing

**Figure 1:**
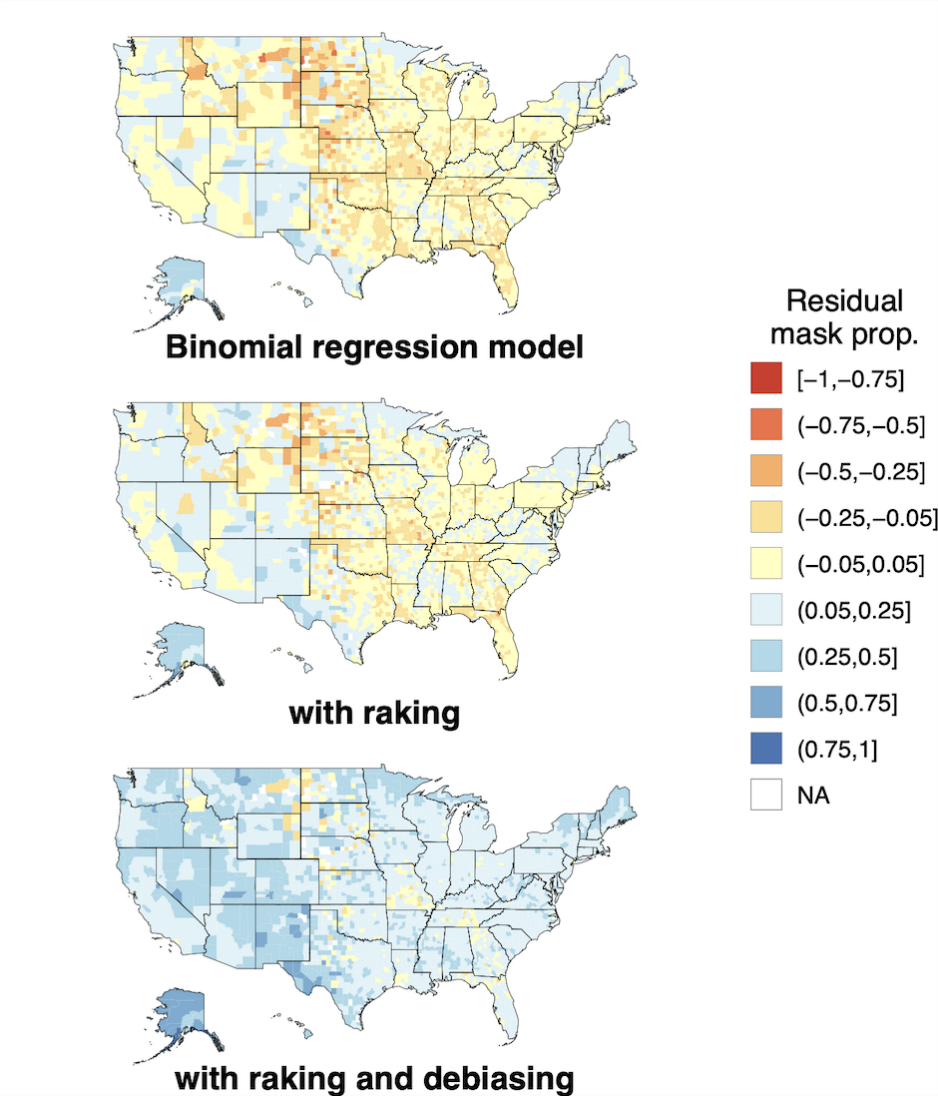
Visualization of spatially heterogeneous data-processing effects. (A) Residuals following the binomial regression model. (B) Residuals following the binomial regression model with raking/sample rebalancing. (C) Residuals following the binomial regression model with raking/sample rebalancing and an offset for bias. Residuals are defined as the difference between the modeled and the observed masking estimates at each analysis stage, where negative values indicate model estimates were higher than observed values and positive residuals indicate model estimates were lower than observed values. All maps are shown for February 2021. N/A: not applicable. See Multimedia Appendix 2 for a high-resolution image.

To demonstrate the spatially heterogeneous effects of our data-processing scheme, Figure 1 shows the difference between estimates from 3 separate models and the raw CTIS masking data. We refer to this difference as the residual, though it is only an indicator of model fit in Figure 1A. In Figures 1B and 1C, the residual values indicate where data corrections caused the largest changes in estimates compared to the original data. After modeling the data with binomial regression, estimates of masking proportions were higher than the observed values (Multimedia Appendix 1, Figure S4) in the central United States and slightly lower than the observed values in the Northeast, Northwest, and Southwest (Figure 1A). Adjusting for unrepresentative samples with raking and resampling and rerunning the binomial regression model had a minor effect on mask-wearing estimates, only exhibiting a slight decrease compared to the model without raking (Figure 1B). Correcting for survey biases using vaccination data in the binomial regression model run on raked survey responses systematically decreased masking proportions, as expected and denoted by increased residuals (Figure 1C). (A map showing the spatial distribution of these biases is given in Multimedia Appendix 1, Figure S19.) We refer to estimates from the model in Figure 1C as debiased or bias-corrected for the remainder of the paper. Our results reinforce that behavioral surveillance should be conducted carefully to limit bias initially.

### Masking Behavior Exhibits Spatial and Temporal Heterogeneity and Is Positively Associated With Population Density

**Figure 2:**
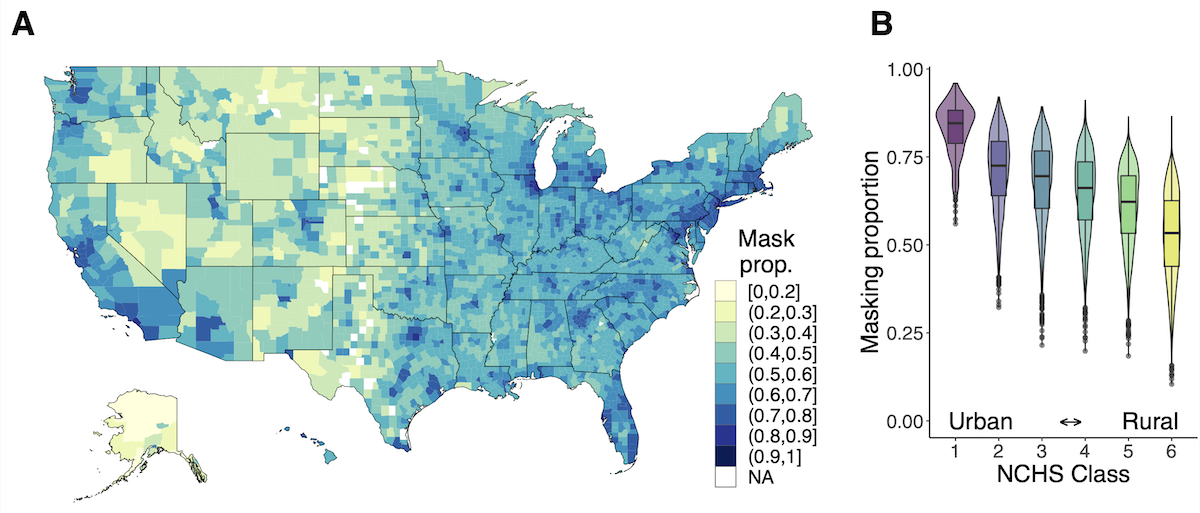
Bias-corrected masking behavior is spatially heterogeneous and higher in urban areas. (A) Map of bias-corrected masking behavior in October 2020 reveals high spatial heterogeneity. Masking proportions vary substantially even within a single state. Spatial heterogeneity does not notably vary over time (Multimedia Appendix 1, Figure S5). A selection of other months in the study period are shown in Multimedia Appendix 1 (Figures S6-S8). (B) Breakdown of county masking proportions over all survey months by the NCHS urban-rural classification. A direct relationship between the median masking proportion and population density is observed. N/A: not applicable; NCHS: National Center for Health Statistics. See Multimedia Appendix 3 for a high-resolution image.

Using bias-corrected masking proportions from the CTIS, we found that masking behavior was spatially heterogeneous over all months (Moran's I between 0.68 and 0.70 for all months, Figures 2A and S5 in Multimedia Appendix 1). Bias-corrected masking proportions ranged from 0.11 to 0.96 and varied substantially within states, emphasizing the importance of analyzing masking behavior at finer scales than the state or Health and Human Services (HHS) region level. Masking proportions were closely linked to population density over the survey period: urban counties tended to have higher masking proportions than rural counties (Figure 2B). Although masking proportions ranged quite a bit within NCHS urban-rural classifications, all differences between NCHS classes were significant (Kruskal-Wallis and pairwise Wilcox test, n=27,842, all *P*<.001). Over all counties and survey months, the median fitted masking proportion in urban counties exceeded 0.8, while the median fitted masking proportion in the most rural counties was below 0.6.

**Figure 3:**
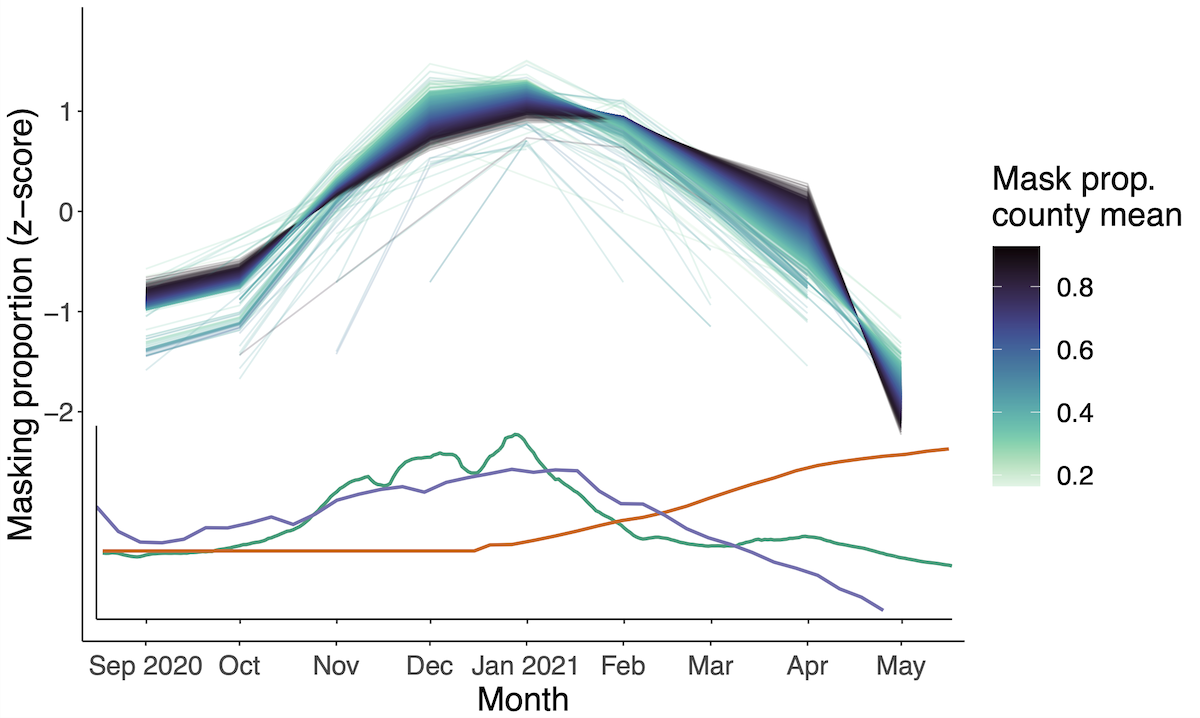
Bias-corrected masking behavior peaked in the winter of 2020-2021 and fell in the spring of 2021, mirroring new cases and increasing vaccinations. Top curves show the time series of the z-score of bias-corrected masking proportions for each county colored by the average masking proportion across the survey period. The inset plot shows z-scores of the 7-day rolling average of new cases (green), the proportion of individuals vaccinated nationally (orange), and the reported worry about severe illness from COVID-19 in CTIS respondents (purple). Z-scores are based on the mean and SD of each county’s masking estimates over the survey period. CTIS: COVID-19 Trends and Impact Survey. See Multimedia Appendix 4 for a high-resolution image.

Masking behavior not only varied geographically but also temporally. Peak masking behavior was observed in January 2021, while the lowest masking proportions were observed in May 2021 (Figure 3). Counties with higher mean masking proportions fluctuated less than counties with lower mean masking proportions from September to April but experienced the largest differences from their mean values in May 2021. For context, we highlight that this decrease coincides with increasing proportions of vaccinated individuals in the United States (Figure 3, [33]), declining new infections [44], and decreasing reported worry about severe illness due to COVID-19 from the CTIS [27]. The policy context during this time was also shifting: On April 27, 2021, the CDC announced that fully vaccinated individuals no longer needed to wear masks outdoors [45], and on May 13, 2021, it announced that fully vaccinated individuals no longer had to wear masks indoors either [46]. Meanwhile, 49% of counties that ever had a mask mandate lifted it before May 1, 2021 (Multimedia Appendix 1, Figure S9). These announcements coincided with the observed decrease in masking in these months. Together, these analyses underscore the importance of tracking and analyzing mask wearing at fine spatial and across long temporal scales: further spatial and temporal aggregation of these data would have missed key heterogeneity previously not quantified and prevented future work from investigating the connection between policy and behavior change at appropriate granularity.

### Community-Reported Masking Levels Are a Good Predictor of Bias-Corrected Self-Reported Estimates

**Figure 4:**
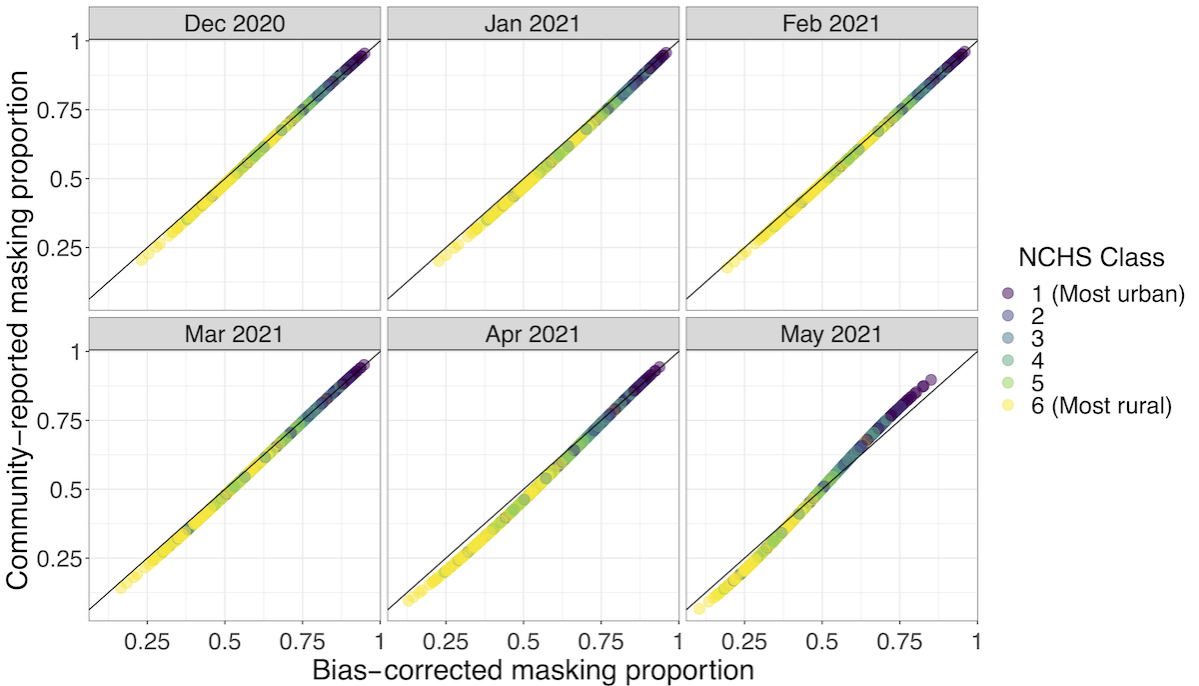
Community-reported masking gives a good estimate of bias-corrected self-reported masking. Community-reported masking refers to the CTIS question where individuals report how many people in their community are masking, which may decrease nonresponse and social desirability biases, compared to asking individuals to self-report their masking behavior. Point color denotes urban-rural classes. Comparisons of individual- and community-reported estimates at different analysis stages are shown in Multimedia Appendix 1 (Figures S11 and S12). CTIS: COVID-19 Trends and Impact Survey. See Multimedia Appendix 5 for a high-resolution image.

Bias-corrected masking proportions were well approximated by modeled estimates of community-reported masking (Figure 4). The difference between the 2 mask-wearing proportion estimates ranged from –6% to 5% and became more apparent in April and May 2021, particularly in rural areas. In May 2021, though, community estimates in urban areas tended to overestimate bias-corrected individual masking estimates. This result is quantitatively affected by influential observations but is qualitatively robust (Multimedia Appendix 1, Figure S10). These results suggest that surveying participants about community masking may give less biased responses than asking individuals to report their own masking behavior, potentially reducing social desirability bias and capturing parts of the population that may be otherwise less likely to respond to the survey.

## Discussion

### Principal Findings

Despite the widespread adoption of face mask-wearing at points during the COVID-19 pandemic in the United States, the true prevalence of this behavior across temporal and spatial scales is largely unknown. Data on mask wearing have been collected through surveys, at varying spatiotemporal resolutions and with potentially varying survey biases (eg, [15-17]). Here, we characterized mask-wearing behavior across the United States using self-reported masking data from a large national online survey. We used Bayesian binomial regression models to remediate issues of small sample size, performed raking/sample balancing to address unrepresentative survey samples, and corrected for additional response biases using measurable bias in vaccination data. We observed substantial spatial heterogeneity in the masking behavior across urban versus rural counties, with some temporal changes in mean masking estimates at the county-month level, most notably a steep decline in masking in May 2021. We found that community-reported masking responses well approximated our bias-corrected masking estimates. Other work adds to this validation: Similar spatial heterogeneity was found in 2 other surveys, with some overlapping periods (Multimedia Appendix 1, Figures S1-S3, [17,47]), and our debiased estimates generally agree with those recorded in observational studies, including higher levels of masking in urban areas (Multimedia Appendix 1, Table S1, [14,28]). Our results reveal the landscape of the masking behavior across 3 distinct phases of the pandemic (presurge, surge during winter 2020-2021, and postsurge during the initial COVID-19 vaccination rollout). Our work also highlights the critical role that behavioral big data can play in the pandemic response, if such data are used with caution.

### Implications

Our findings have several implications for the fields of infectious disease epidemiology and public health policy. We identified high spatial and moderate temporal heterogeneity in masking behavior at the county-level—patterns that are obscured if data are aggregated to the state or HHS region level. Contrary to our expectations, this level of spatial variability around the mean is consistent over time. Consequently, disease models should account for spatial variability in masking behavior but may only need to consider changes in masking dynamics over longer temporal scales. The high spatial heterogeneity we found in masking behavior also highlights the need for diverse and targeted public health approaches across the country rather than a single national program. Guidance set at the state level without regard for differences in local conditions may miss early opportunities to control disease spread, prematurely enforce public health restrictions, and contribute to fatigue with public health restrictions. Thus, we advocate for local behavioral data collection and geographically targeted public health policy for optimized resource use and efficient disease suppression.

Although county-level mask-wearing behavior varied across months, we observed little heterogeneity across counties in these temporal trends. The observed changes in masking behavior roughly correspond to national trends in new cases in the United States and self-reported worry about severe disease, as reported in the CTIS, though we did not determine causality or examine this relationship at the individual or county level. Because we modeled county-level averages, this observed correlation could be driven by a specific demographic group or subset of individuals modifying their masking behavior, rather than a uniform change in average mask uptake in a county’s population. The sharp decrease in masking in May 2021 is contemporaneous with many states lifting mask mandates (Multimedia Appendix 1, Figure S9) and an announcement from the CDC that vaccinated individuals no longer have to wear masks outdoors (April 27, 2021 [45]) or indoors (May 13, 2021 [46]). It is plausible that these policy changes could have impacted masking behavior, both in vaccinated and in unvaccinated individuals, even though the change in CDC guidance did not apply to unvaccinated individuals [42,48]. More work is needed to explore the potential differences in mask wearing between vaccinated and unvaccinated individuals. Additional research could also focus on quantifying the impact of social norms on individuals’ masking behavior at fine spatiotemporal resolution in the United States.

Recent work has highlighted the potential for big data sources to provide a measurement of spatially disaggregated social phenomena (eg, [49]), while other research points to the challenges of inferring high-quality estimates of behavior from such high-volume data sources [19]. In our work, we sought to steer away from “big data hubris” [50] by applying rigorous statistical methods to manage concerns about representativeness and bias and by conducting an internal validation of our model-based estimates [51]. In particular, we addressed representativeness by age, sex, and education to capture sociodemographic response bias. Motivated by an association between COVID-19–preventative behaviors [39], we debiased our masking estimates based on vaccination data to address additional nonresponse bias and social desirability bias. Finally, we internally validated our population-scale masking estimates of self-reported behaviors with responses of community behaviors, which may be subject to less nonresponse and social desirability bias compared to self-reporting questions [52]. We found that community-reported masking estimates agreed closely with bias-corrected self-reported masking behavior, highlighting that surveying participants about community behavior may be an avenue to reduce survey bias. We note, however, that this finding may not apply in all settings; self-reported masking behavior on a university campus closely matched observed masking levels, and questions about community masking were less accurate [53]. Although these implications for analysis of surveys on human behavior may not apply universally, similar results have been found in other infectious disease applications, including disease surveillance (using the CTIS data [27,54]) and early outbreak detection in social networks [55,56]. Our results further emphasize the promise of human social sensing going forward to make big data sources more meaningful [57].

### Limitations

Nevertheless, our approach has some limitations that are important to consider. We were unable to deal with all representation or response biases, including the exclusion of individuals under 18 years of age; a lack of representativeness due to factors other than age, sex, and education; recall bias; dishonest responses; and other characteristics that may be predictive of nonresponse or social desirability bias, such as political leanings or belief in COVID-19 conspiracies [27]. Likewise, we could not account for how individuals with Facebook accounts may engage differently in COVID-19–preventive behaviors than non-Facebook users. It is unclear whether these biases that are unaccounted for would have a systematic or random effect on our results. However, we do expect their impact to be relatively small, particularly as our ground-truth–based debiasing approach adjusts masking estimates regardless of the source of nonresponse bias, and as supported by our community-reported masking analysis (since the community question captures some of the non-Facebook user population). We assumed that self-reported mask wearing is biased in the same magnitude and direction as the self-reported COVID-19 vaccination status—an assumption that should be tested in future research. Our approach also does not resolve issues of a small sample size; for example, the association we found between bias-corrected self-reported masking and community-reported masking is stronger between modeled estimates than between raw means. Although we attempted to correct for bias in our mask-wearing estimates, the point estimates for these values should be interpreted with caution. The goal of our work is not to produce point estimates of county-level mask-wearing behavior but instead to take advantage of the CTIS survey design and characterize the relative trends in mask-wearing behavior between and across counties. We advocate for additional observational studies with experimental designs that would allow for direct estimation of these quantities to improve behavioral surveillance estimates.

### Conclusion

In summary, we produced the first accurate high-resolution spatiotemporal estimates of face mask wearing in the United States for the period from September 2020 through May 2021. Our work reveals that masking behavior is highly variable across the United States, suggesting that a one-size-fits-all approach to increasing mask-wearing behavior is likely to be ineffective. Instead, we identified regions of the country with higher and lower masking levels. These differences should be investigated going forward as public health organizations consider how to more effectively target these low-masking regions. For example, these communities may be more susceptible to mis- and disinformation regarding mitigation behaviors, which must be strategically confronted. Furthermore, this variability in behavior demonstrates the need to develop infectious disease dynamics models to analyze and predict how spatiotemporal trends in disease are affected by changes in human behavior, such as vaccination, contact patterns, and face mask wearing. Our analyses also address issues of survey bias, with the takeaway that, in the future, we should invest in a robust survey infrastructure that can recruit large representative samples with minimal bias, including using certain representative respondents as human social sensors to report on their communities.

## Appendix

Multimedia Appendix 1 Supplementary materials.

Multimedia Appendix 2 Visualization of spatially heterogeneous data-processing effects. (A) Residuals following the binomial regression model. (B) Residuals following the binomial regression model with raking/sample rebalancing. (C) Residuals following the binomial regression model with raking/sample rebalancing and an offset for bias. Residuals are defined as the difference between the modeled and the observed masking estimates at each analysis stage, where negative values indicate model estimates were higher than observed values and positive residuals indicate model estimates were lower than observed values. All maps are shown for February 2021. N/A: not applicable.

Multimedia Appendix 3 Bias-corrected masking behavior is spatially heterogeneous and higher in urban areas. (A) Map of bias-corrected masking behavior in October 2020 reveals high spatial heterogeneity. Masking proportions vary substantially even within a single state. Spatial heterogeneity does not notably vary over time (Multimedia Appendix 1, Figure S5). A selection of other months in the study period are shown in Multimedia Appendix 1 (Figures S6-S8). (B) Breakdown of county masking proportions over all survey months by the NCHS urban-rural classification. A direct relationship between the median masking proportion and population density is observed. N/A: not applicable; NCHS: National Center for Health Statistics.

Multimedia Appendix 4 Bias-corrected masking behavior peaked in the winter of 2020-2021 and fell in the spring of 2021, mirroring new cases and increasing vaccinations. Top curves show the time series of the z-score of bias-corrected masking proportions for each county colored by the average masking proportion across the survey period. The inset plot shows z-scores of the 7-day rolling average of new cases (green), the proportion of individuals vaccinated nationally (orange), and the reported worry about severe illness from COVID-19 in CTIS respondents (purple). Z-scores are based on the mean and SD of each county’s masking estimates over the survey period. CTIS: COVID-19 Trends and Impact Survey.

Multimedia Appendix 5 Community-reported masking gives a good estimate of bias-corrected self-reported masking. Community-reported masking refers to the CTIS question where individuals report how many people in their community are masking, which may decrease nonresponse and social desirability biases, compared to asking individuals to self-report their masking behavior. Point color denotes urban-rural classes. Comparisons of individual- and community-reported estimates at different analysis stages are shown in Multimedia Appendix 1 (Figures S11 and S12). CTIS: COVID-19 Trends and Impact Survey.

## Abbreviations

CDC: Centers for Disease Control and Prevention
CTIS: COVID-19 Trends and Impact Survey
HHS: Health and Human Services
NCHS: National Center for Health Statistics

## Data Availability

Data aggregated to the county-month scale and all code to analyze these data will be made available on GitHub [58]. Individual survey responses cannot be shared by the authors, but researchers can refer to Ref. [59] if they would like to enter an agreement for data usage with the Carnegie Mellon University (CMU) Delphi team.
