# Supplementary material for: Spatiotemporal Trends in Self-Reported Mask-Wearing Behavior in the United States: Analysis of a Large Cross-sectional Survey
Source: JMIR Public Health Surveill. 2023 Mar 6;9:e42128. doi: 10.2196/42128 (PMC10028521; doi:10.2196/42128)
Supplement: Multimedia Appendix 1 [file publichealth_v9i1e42128_app1.pdf]

Supplementary Materials

Additional CTIS details

The COVID-19 Trends and Impact Survey was created by researchers in the Delphi Group at Carnegie Mellon University and distributed via Facebook to active users 18 or older starting in April 2020. On a daily basis, a random state-stratified sample of Facebook users are invited to take the survey at the top of their news feed. These users will not be re-invited to take the survey for at least thirty days. The survey asks a broad range of questions related to COVID-19 symptoms and behaviors, with variations across each of the thirteen different waves spanning from April 2020 to June 2022. In this study, we make use of data from Wave 4 (September 8, 2020 - November 23, 2020), Wave 5 (November 24, 2020 - December 18, 2020), Wave 6 (December 19, 2020 - January 11, 2021), Wave 7 (January 12, 2021 - February 7, 2021), Wave 8 (February 8, 2021 - March 1, 2021), and Wave 10 (March 2, 2021 - May 19, 2021, Wave 9 was skipped for numbering purposes).

Weights were provided by Facebook for each response to adjust for non-response and coverage bias at the daily-state level [60]. Briefly, each weight describes the number of people represented by a respondent based on their age, gender, location, and date of response. These weights were calculated via a two-step process using inverse propensity score weighting based on respondents' demographics recorded in their Facebook user profiles to adjust the survey sample to reflect active Facebook users, followed by revisions of these weights using post-stratification so that the survey sample reflects the general population [27]. Because these weights were not at the same scale of our data analysis, we did not use them and instead performed raking to calculate our own weights as described in Methods.

As part of our data processing, we also dropped responses missing fips codes or outside the 50 states and the District of Columbia. When raking, we use three categories for age (18-24, 25-54, and 55+), two categories for sex (ACS)/gender (CTIS) (male and female), and six categories for education (less than high school, high school graduate or equivalent, some college, 2 year college degree, 4 year college degree, and postgraduate degree). Subsetting age further results in issues of nonconvergence. Raking on race/ethnicity with more than two categories leads to substantial nonconvergence whether or not we include education. We drop responses missing age, gender, or education from being raked.

In addition, when dichotomizing CTIS responses we assume that people who "sometimes" wore a face mask in public were not masking which may lead us to underestimate self-reported masking levels. We believe the effects of this assumption are minimal because the proportion of "sometimes" responses was small compared to other options (Figs. S14).

Estimation of CTIS bias

We compare CTIS responses about vaccination to true vaccination estimates for the period from April 1, 2021 through May 31, 2021. We chose this period when nearly all adults were eligible for vaccination in the U.S. so that the sample population responding to the masking and vaccination questions would be most similar. In this time period, the differences between the survey and ground-truth vaccination data have also stabilized (Fig. S18). We use a (frequentist) binomial generalized linear mixed-effects model to estimate p_i, the proportion of respondents who were vaccinated at the county-level each week. If V_i is the number of (partially) vaccinated respondents in each county i out of N_i respondents, then the model is as follows:

$$V_i \sim Binomial(N_i, p_i)$$
$$\text{logit}(p_i) = \beta_0 + \beta_1 t + \beta_2 t^2 + u_i$$
$$u_i \sim Normal(0, \sigma_u^2)$$

where t and t^2 are orthogonal polynomials of degree 1 and 2, respectively, generated by the `poly` function in R from the rank of the weeks in which the vaccination data were observed. Therefore, β_1 and β_2 are covariates that describe the trend of time in expected reported vaccination across counties, while u_i describes systematic difference in vaccination in county i relative to the mean trend. This model was implemented using `glmer` in the `lme4` package [36].

Using these modeled CTIS county-level vaccination proportions, we compared them with the true vaccination data to calculate the expected bias in reported survey responses relative to ground truth data in county i. There were 45 counties that had missing bias estimates due to either a lack of weekly CTIS vaccination survey responses between 0 and 1 (cannot use logits of $p = 0, 1$), or missing true vaccination estimates for weeks with survey responses between 0 and 1 (therefore, cannot calculate difference between true and CTIS vaccination estimates).

CTIS model coefficients

Model coefficients for z-score($\log_{10}$(population density)) ranged from 0.45 to 0.55 (Fig. S22), meaning a one unit change in z-score($\log_{10}$(population density)) is correlated with the expected odds of masking multiplying by $e^{0.5} \approx 1.65$. The coefficient of the population density covariate in our binomial regression model is consistent over time, indicating that the relationship between population density and masking behavior is stable across months.

CTIS mixed effects model specifications

In addition to the models presented in the main text, we ran two models to estimate mask-wearing at the county-month level using state or county-level random effects. For both models, we define M_i as the number of respondents masking in county i (e.g., respondents that masked most or all of the time in the past 5-7 days), N_i as the total number of respondents in county i ($M_i \leq N_i$), and p_i as the county-level probability of a response consistent with masking. We ran both models using `brms` [29] with the `cmdstanR` [30] backend and we ran the sampler with 4 chains for 3000 iterations per chain. We use the following model to estimate $\hat{p}_i$ and $\hat{M}_i$ with state-level effects:

$$
\begin{aligned}
M_i &\sim Binomial(N_i, p_i) \\
\text{logit}(p_i) &\sim Normal(\mu_i, \sigma) \\
z_i^D &= \left(\frac{D_i - \bar{D}}{\sigma_D}\right) \\
\mu_i &= \beta_0 + \beta_1 * z_i^D + state_i \\
\beta_0 &\sim Normal(0, 1) \\
\beta_1 &\sim Normal(1, 1) \\
state_i &\sim Normal(0, \sigma_{state}) \\
\sigma_{state} &\sim t_3(0, 2.5)
\end{aligned}
$$

where $D_i = \log_{10}(\texttt{population density}_i)$ for county i. The $\hat{R}$ values were frequently ≥ 1.02 and $n_{eff} < 500$ for the intercept population-level effect and group-level effects, indicating lack of convergence. The population density coefficient did show convergence ($\hat{R} = 1$ and $n_{eff} > 1800$) and remained consistent over time and close to the values observed in the original model. Only approximately 1% of observations had Pareto k values > 0.7 indicating that the model was robust to the influence of individual observations.

For county-level effects we used the following model to estimate $\hat{p}_i$ and $\hat{M}_i$:

$$
\begin{aligned}
M_i &\sim Binomial(N_i, p_i) \\
\text{logit}(p_i) &\sim Normal(\mu_i, \sigma) \\
z_i^D &= \Big(\frac{D_i - \bar{D}}{\sigma_D}\Big) \\
\mu_i &= \beta_0 + \beta_1 * z_i^D + fips_i \\
\beta_0 &\sim Normal(0, 1) \\
\beta_1 &\sim Normal(1, 1) \\
fips_i &\sim Normal(0, \sigma_{fips}) \\
\sigma_{fips} &\sim t_3(0, 2.5)
\end{aligned}
$$

where $D_i = \log_{10}(\texttt{population density}_i)$ for county i. In this case sampler diagnostics indicated reasonable model convergence with $\hat{R} \leq 1.01$ and $n_{eff} > 600$ for all months except April and May 2021 when $n_{eff} > 250$. However, over 50% of observations in each month had Pareto k values > 0.7, indicating the influence of each data point and potential overfitting. As observed with the state-level random effects model, the population density coefficient remained consistent over time with values near those from the original model.

Outbreaks Near Me survey details & results

The Outbreaks Near Me (ONM) survey was created by scientists at Harvard and Boston Children's Hospital and distributed through a partnership with SurveyMonkey. Following the completion of another survey on SurveyMonkey, a random representative sample of users across the United States were invited to take the Outbreaks Near Me survey. The survey was released in June 2020 and asked respondents how likely they were to wear a mask in several different environments: while grocery shopping, visiting with friends and family in their homes, exercising outside, and in the workplace. Answer options were (1) Very, (2) Somewhat, (3) Not so, or (4) Not likely at all (Fig. S23). We focus on the responses to the grocery shopping scenario, as this setting is most comparable to the "in public" scenario described in the CTIS question. To dichotomize these responses for an analysis of the proportion of respondents wearing masks, we consider "very likely" responses as masking and all other responses as not masking. This choice allows for comparison with CTIS data and makes sense given the small percentage of "somewhat likely" responses (Fig. S24). We aggregate responses at the zipcode-month scale and crosswalk these estimates to the county-month level using HUD files [61]. The Outbreaks Near Me survey dropped responses from individuals who reported their age as less than 13 or more than 100 years old. We additionally drop respondents who did not respond to the grocery store portion of the masking question or who have an invalid zip code that cannot be crosswalked to a fips code; this process leaves us with 1,042,685 valid responses. Survey weights were provided for individual responses at the weekly-state and daily-national scales, though we do not to use them because our analysis focuses on the county-month scale.

Due to small sample sizes, we use binomial regression models to estimate masking proportions for each county-month, as described in 'Bayesian binomial regression model' in the Methods (Figs. S25, S26). We compare estimates from these models to CTIS values calculated using only the binomial regression model, i.e., no raking/resampling or debiasing. We calculate the ratio of ONM to CTIS masking proportions for each county each month and take the average across all months in the survey, excluding counties that have estimates for fewer than five of nine months. Additionally, we visualize the time series of individual counties from the two surveys side by side.

Supplemental References

60. Meta. User Guide for the COVID-19 Trends and Impact Survey Weights; 2022. Version 1.

61. U S Department of Housing and Urban Development. HUD USPS ZIP Code Crosswalk Files; 2021. https://www.huduser.gov/portal/datasets/usps_crosswalk.html.

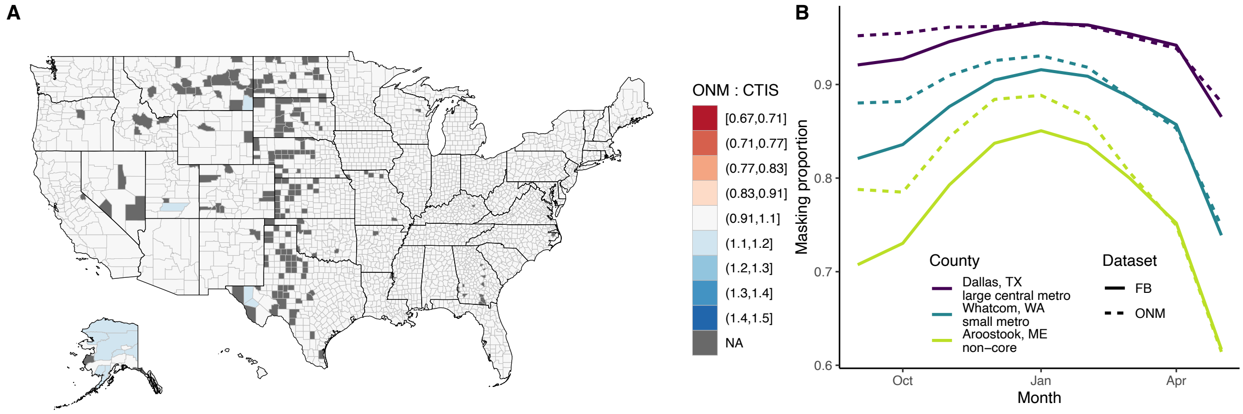

Figure S1. CTIS and ONM responses show similar values and trends across space and time. (A) Average ratio of ONM to CTIS masking proportions for each county from September 2020 through May 2021. All average ratios are greater than 1, but only 0.4% are greater than 1.1. Counties with estimates for less than five of nine months are excluded. (B) Across three counties of varying urbanicity, masking proportions increase through January 2021 and then decrease through May 2021 in both surveys. Differences between survey estimates appear to decrease over time. Urban counties may exhibit higher levels of masking and less variability in these estimates over time compared to rural counties. Both CTIS and ONM estimates are outputs from binomial regression models without raking or bias offsets.

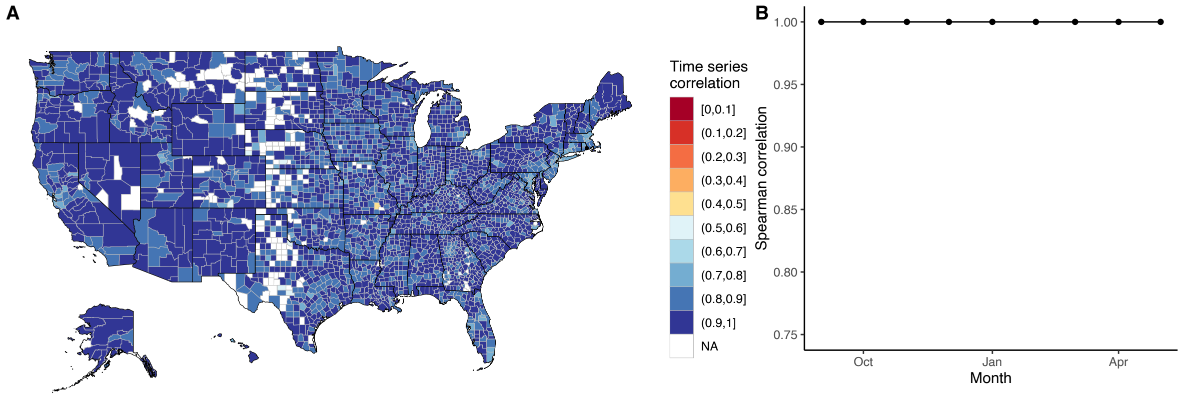

Figure S2. Time series correlations between the CTIS and ONM surveys. (A) Time series correlation for each county across all months is high across all counties. (B) Time series correlation for all counties each month is 1 across all months. Both CTIS and ONM estimates are outputs from binomial regression models without raking or bias offsets.

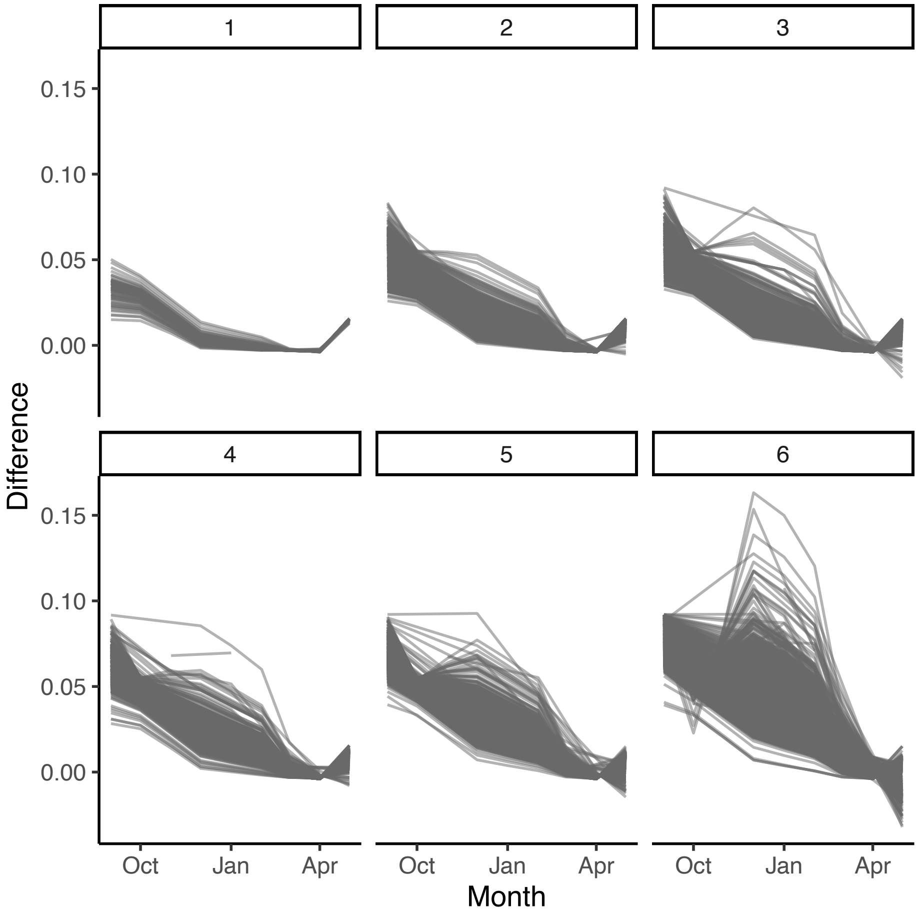

Figure S3. Difference between county masking estimates from ONM and CTIS surveys decreases over time, with greater variability in more rural counties. Facets designate NCHS urban-rural classification with 1 being the most urban and 6 the most rural. Difference between survey estimates goes to nearly 0 across all counties in April 2021, with increasing, albeit small, differences in May 2021. Both CTIS and ONM estimates are outputs from binomial regression models without raking or bias offsets.

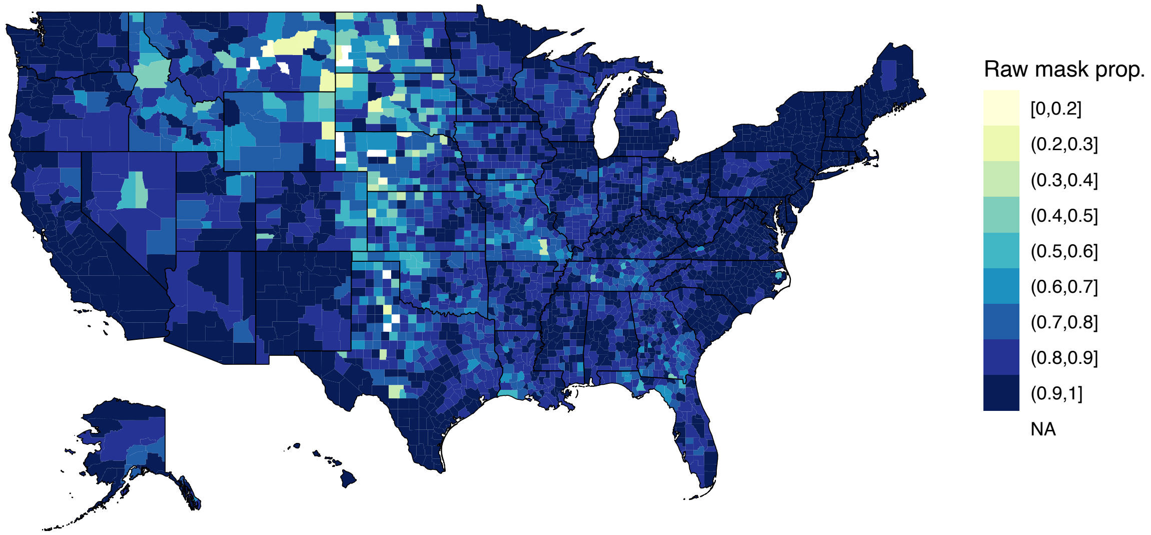

Figure S4. Raw CTIS masking data for February 2021. Proportions masking most of the time or more are much higher than observational studies would suggest, with spatial trends difficult to identify.

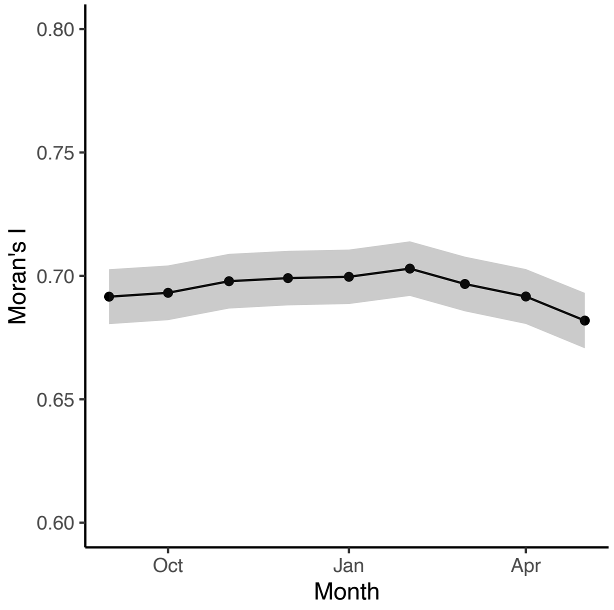

Figure S5. Spatial heterogeneity in self-reported masking behavior does not vary over time. Moran's I calculated for self-reported masking behavior from the CTIS estimates from binomial regression model with raking/resampling and bias offset.

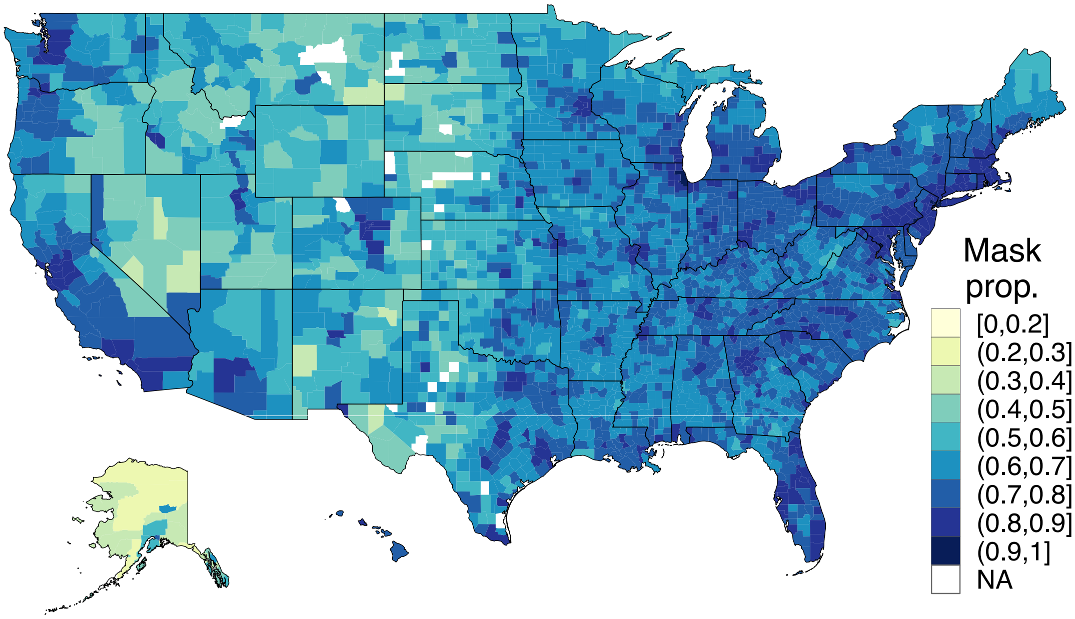

Figure S6. Bias-corrected masking estimates for December 2020.

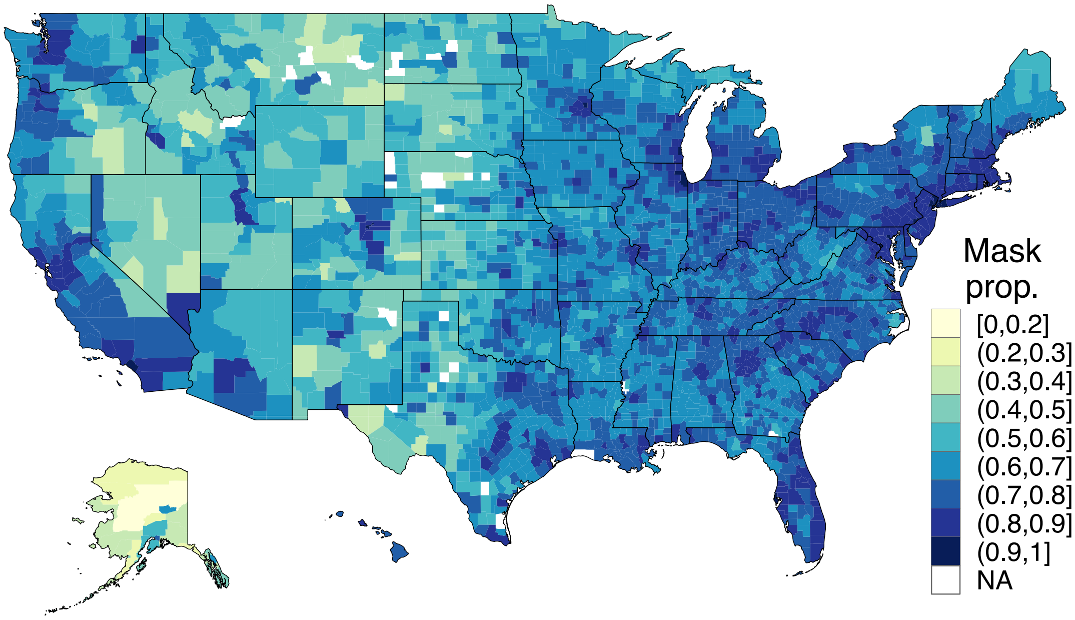

Figure S7. Bias-corrected masking estimates for February 2021.

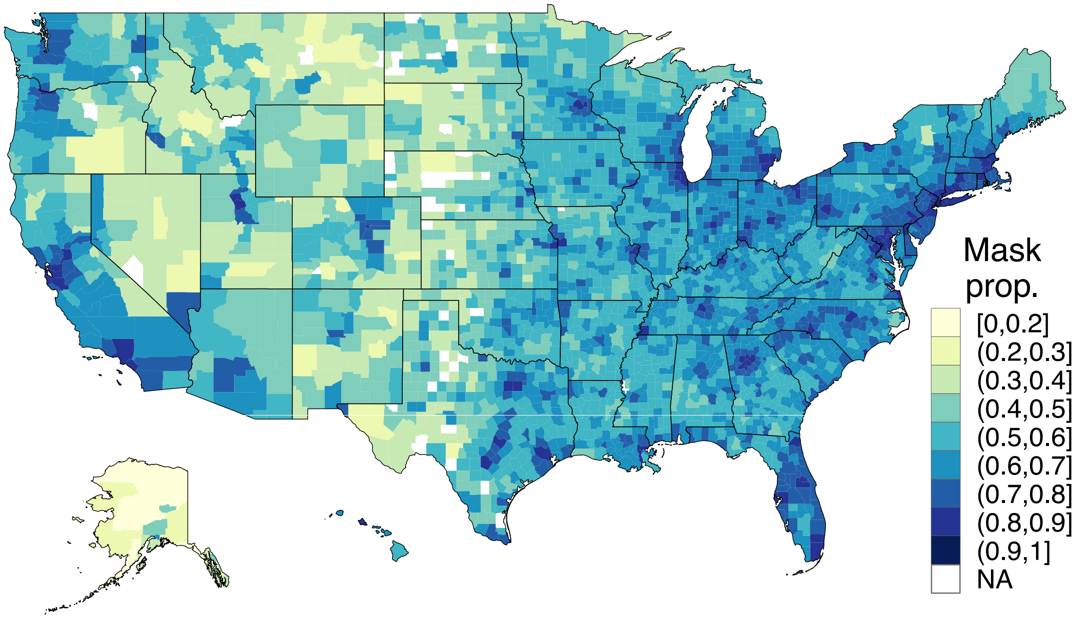

Figure S8. Bias-corrected masking estimates for April 2021.

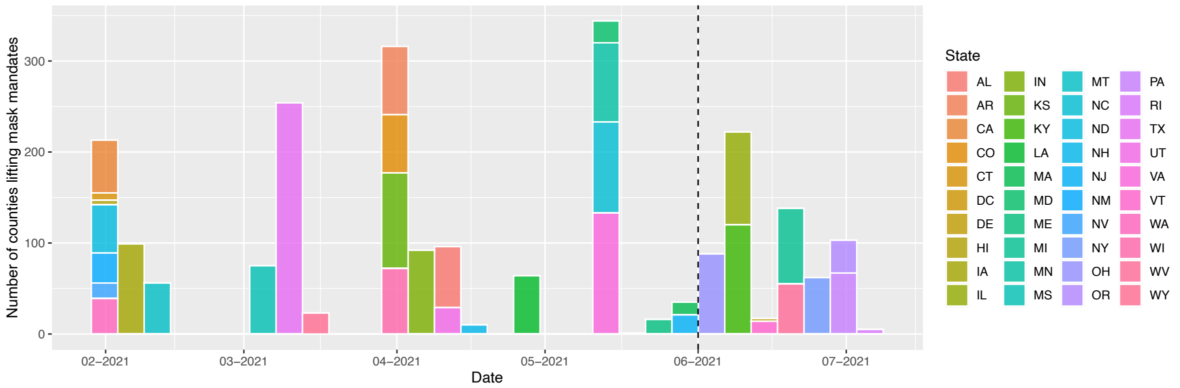

Figure S9. Timeline of lifting of county mask mandates in the U.S. Neither state nor county mask mandates were ever imposed in AK, AZ, FL, GA, ID, MO, NE, OK, SC, SD, and TN during the period from April 10, 2020 to August 15, 2021. Thus, these states have been excluded. Approximately 49% of counties that ever imposed a mask mandate lifted it before May 1, 2021.

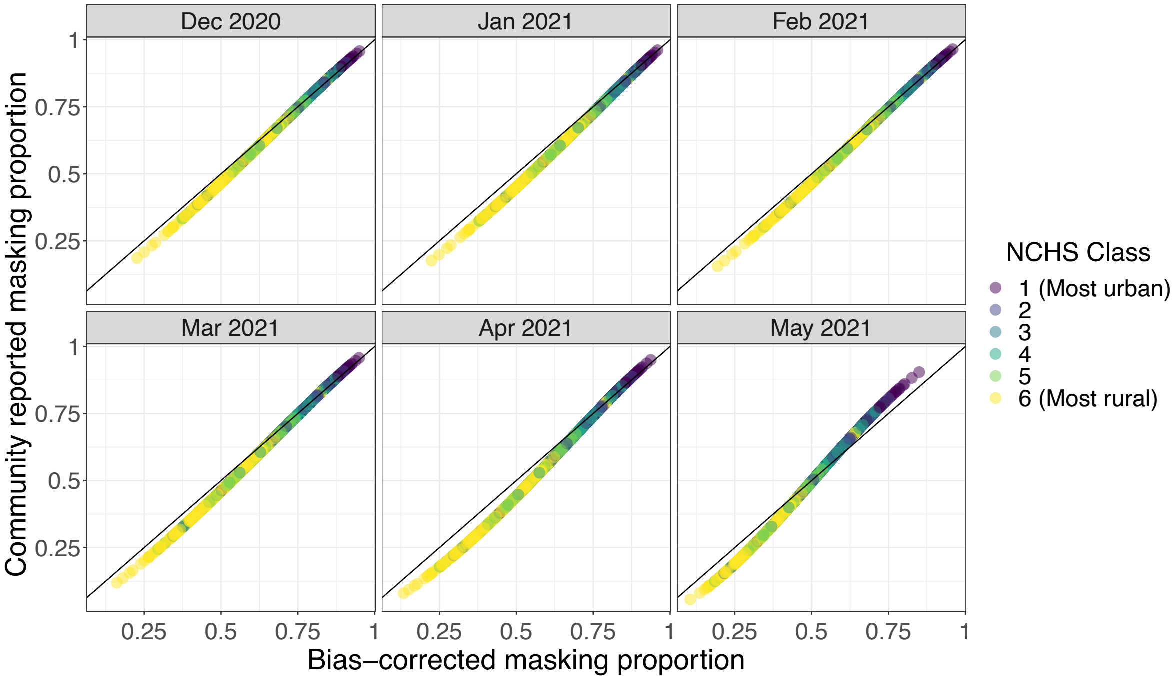

Figure S10. Community reported masking gives a good estimate of bias-corrected self-reported masking even when influential fips code are removed from the model. Fips codes with pareto k values >= 0.7 were excluded from the Bayesian binomial regression model with bias offsets (specifically fips 4019, 6037, 6071, 12071, 12103, 36005, 40143, 41039, 45045, 48201, 48439, 53033). Community reported masking refers to the CTIS question where individuals report how many people in their community are masking, which may decrease non-response and social desirability bias compared to asking individuals to self-report their masking behavior. Point color denotes urban-rural classes.

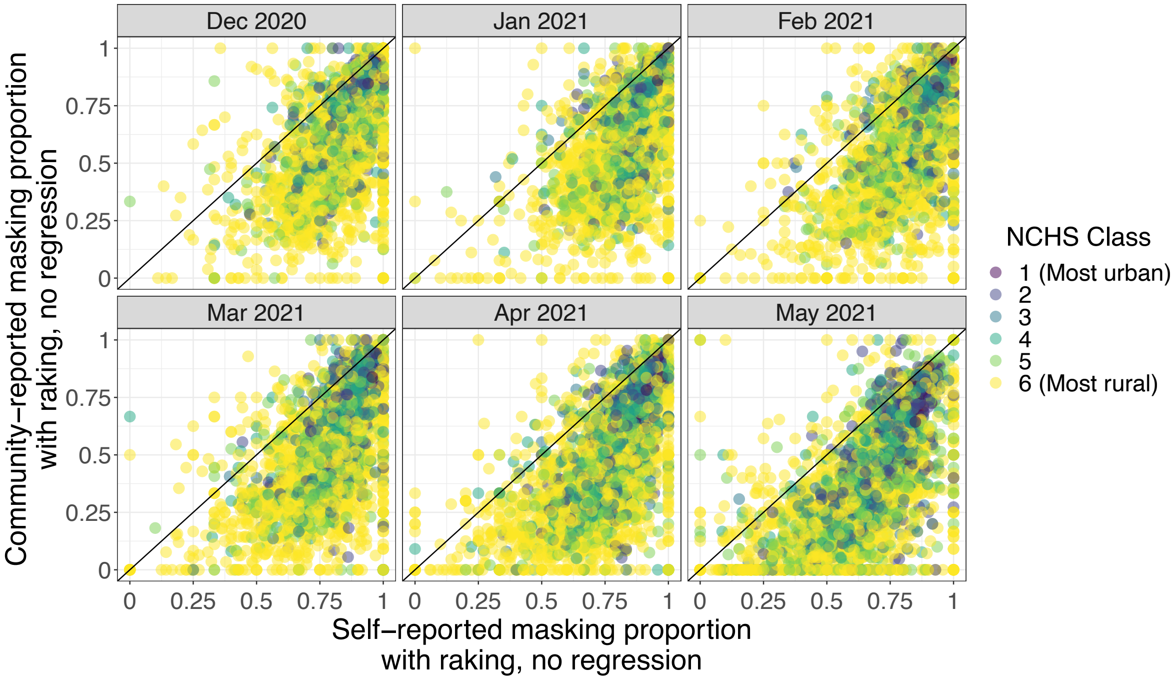

Figure S11. Self-reported masking estimates generally exceed community-reported estimates without bias-correction, but the data are noisy without the binomial regression model. Both masking estimates are calculated from raked and resampled observations but are not run through a binomial regression model to correct for small sample size. Additionally, self-reported masking estimates are not debiased. Recall that community reported masking refers to the CTIS question where individuals report how many people in their community are masking, which may decrease non-response and social desirability bias compared to asking individuals to self-report their masking behavior. Point color denotes urban-rural classes.

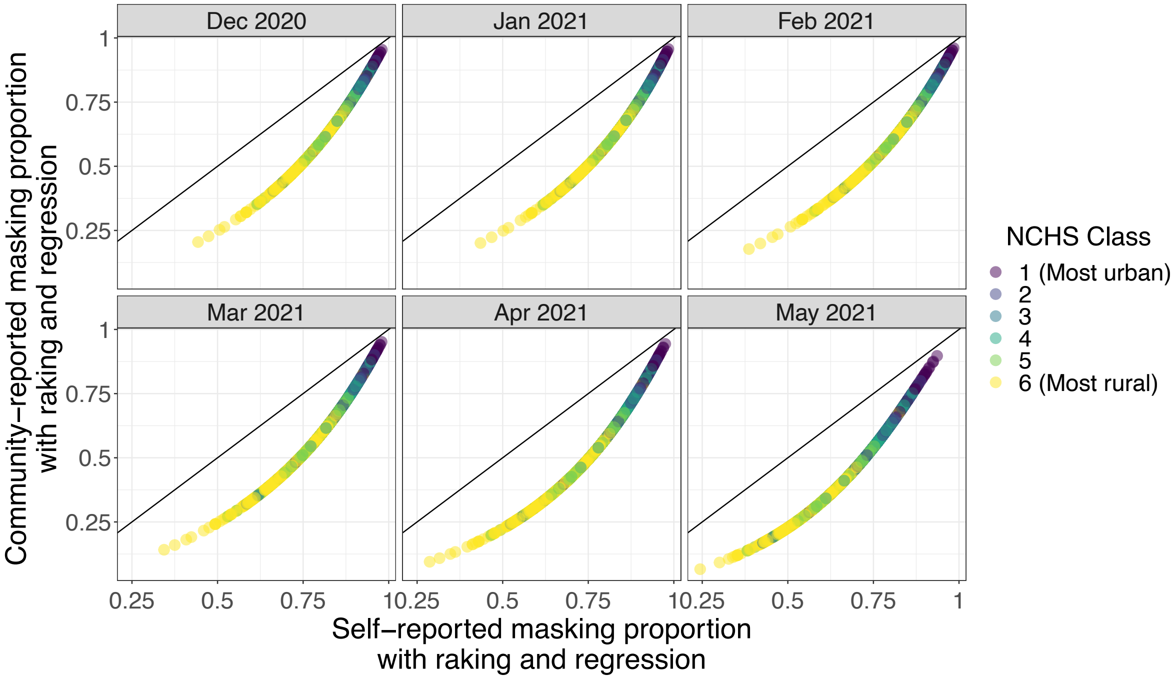

Figure S12. Non-bias-corrected self-reported masking estimates exceed community-reported estimates to a greater degree in rural counties. Both masking estimates are calculated from raked and resampled observations run through a binomial regression model to correct for small sample size. Self-reported masking estimates are not debiased. Recall that community reported masking refers to the CTIS question where individuals report how many people in their community are masking, which may decrease non-response and social desirability bias compared to asking individuals to self-report their masking behavior. Point color denotes urban-rural classes.

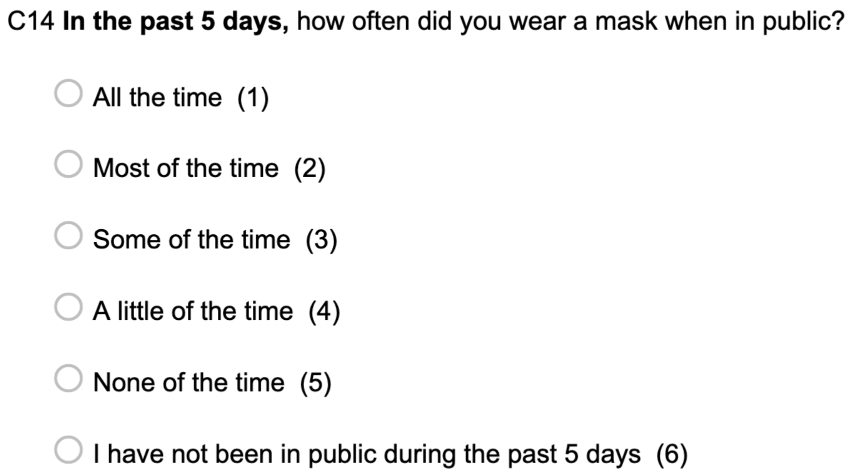

C14 **In the past 5 days,** how often did you wear a mask when in public?

- All the time (1)
- Most of the time (2)
- Some of the time (3)
- A little of the time (4)
- None of the time (5)
- I have not been in public during the past 5 days (6)

Figure S13. CTIS question on masking behavior. Note that time period of interest changed from 5 days to 7 days on February 8, 2021.

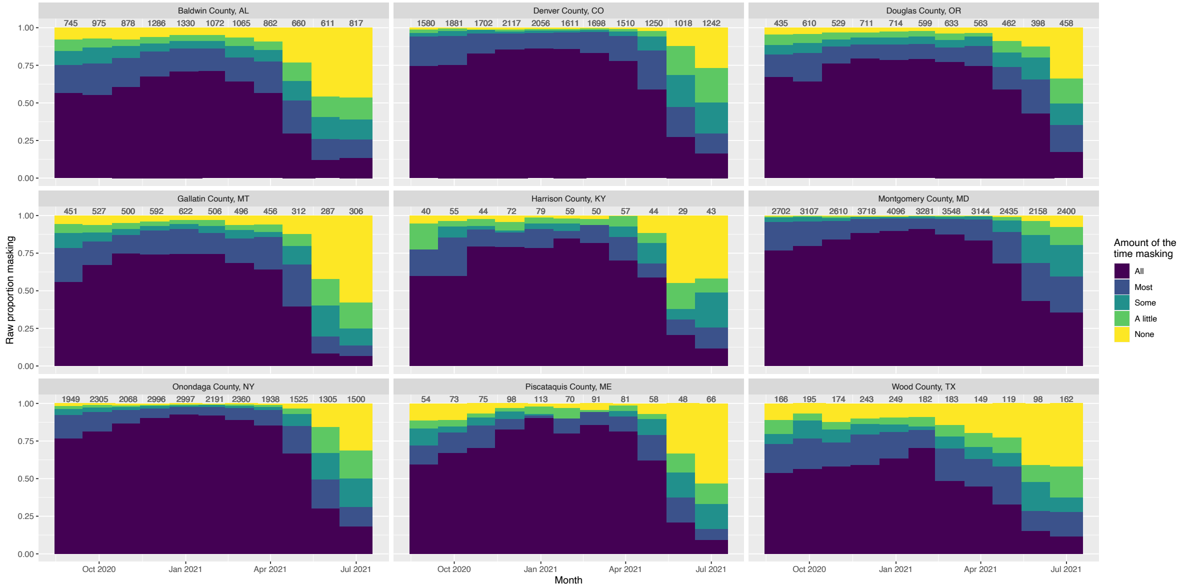

Figure S14. Raw proportions of each CTIS response for a selection of counties spanning population densities across time. Number of responses in that county-month listed above each bar. NCHS urban-rural classifications are as follows: (1) Denver, CO; (2) Montgomery, MD; (3) Onondaga, NY; (4) Baldwin, AL; (5) Gallatin, MT; Douglas, OR; (6) Wood, TX; Piscataquis, ME; Harrison, KY.

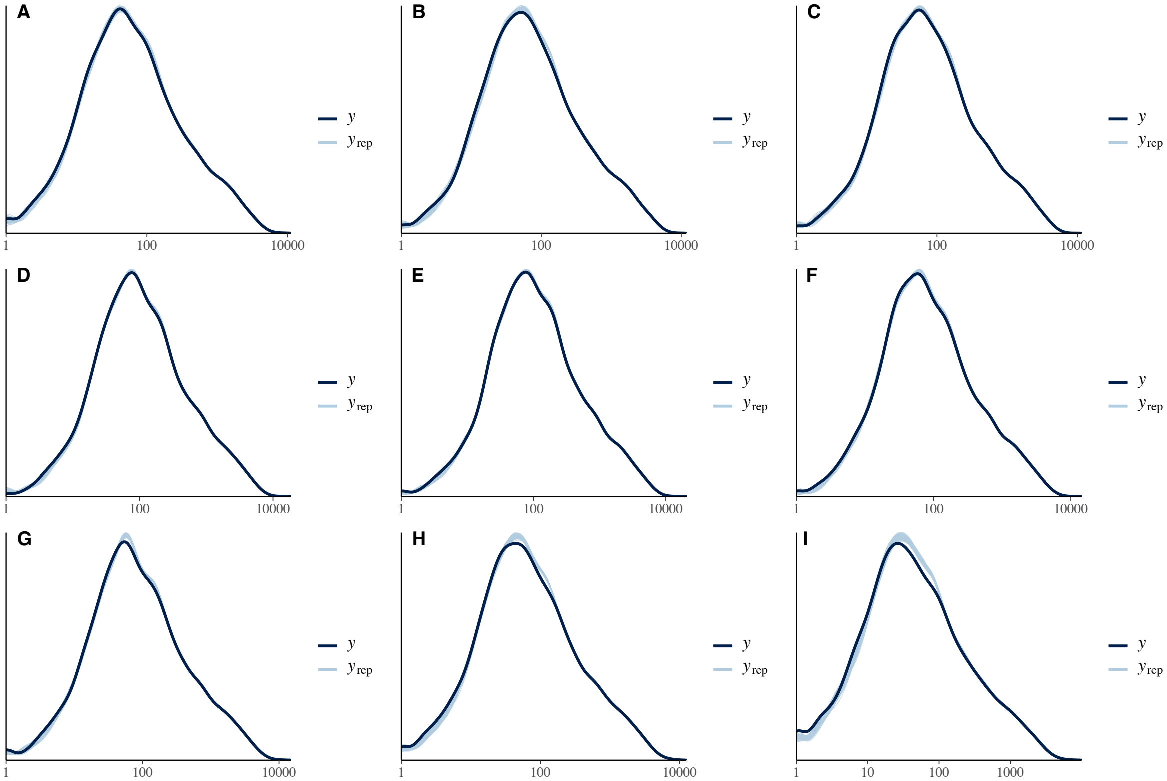

Figure S15. Posterior predictive checks of binomial regression model of CTIS data (no raking or bias offset) with x-axis on a log-10 scale for visual aid. These figures compare observed data (y, black) to model predictions (y_{rep}, blue). The predictions well-approximate the observed data with no systematic differences indicating that the model fit is reasonable.

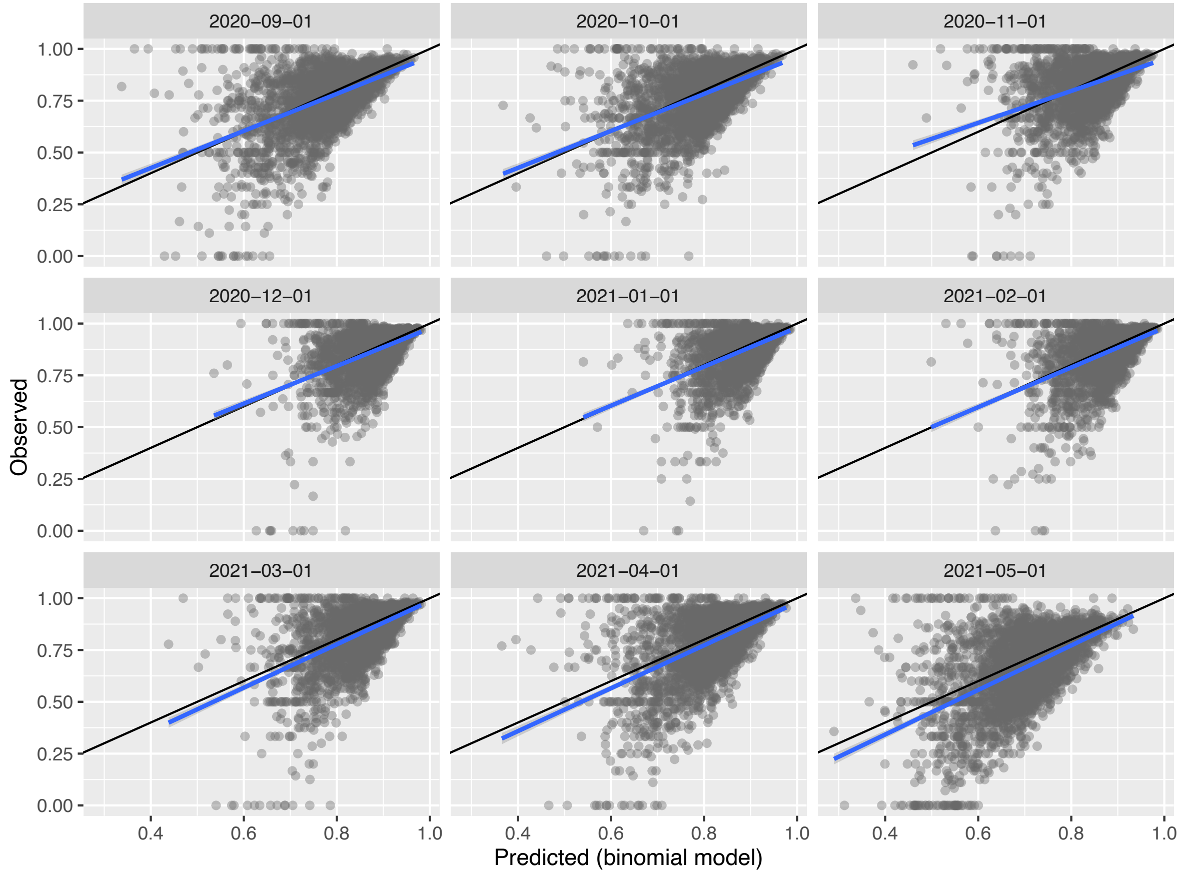

Figure S16. Observed versus predicted CTIS masking estimates with binomial regression model (no raking or bias offset). Black line shows y = x; blue line is a linear fit of the data. The linear trend is close to the one-to-one line indicating good model fit, though there is substantial noise.

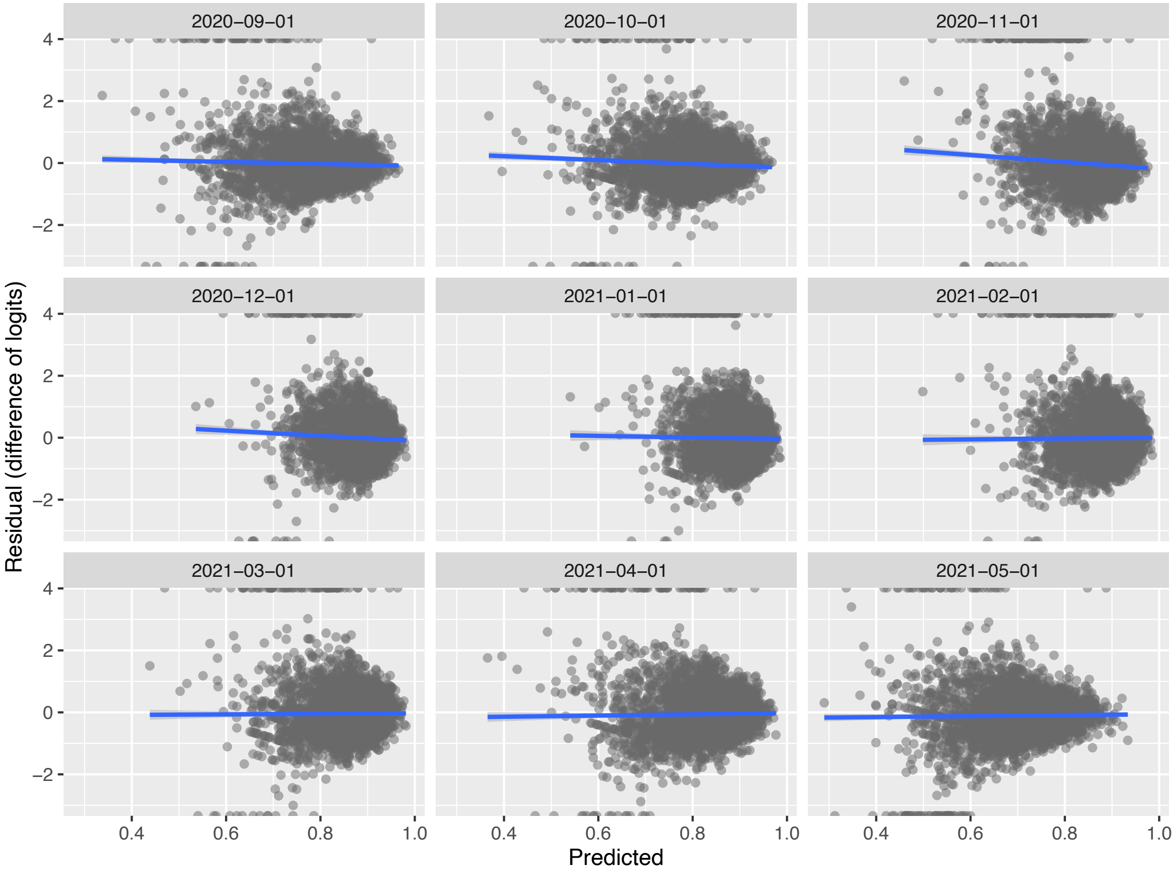

Figure S17. Observed versus residual CTIS masking estimates with binomial regression model (no raking or bias offset). Blue line is a linear fit of the data. Residuals show little association with predicted value which indicates a reasonable model.

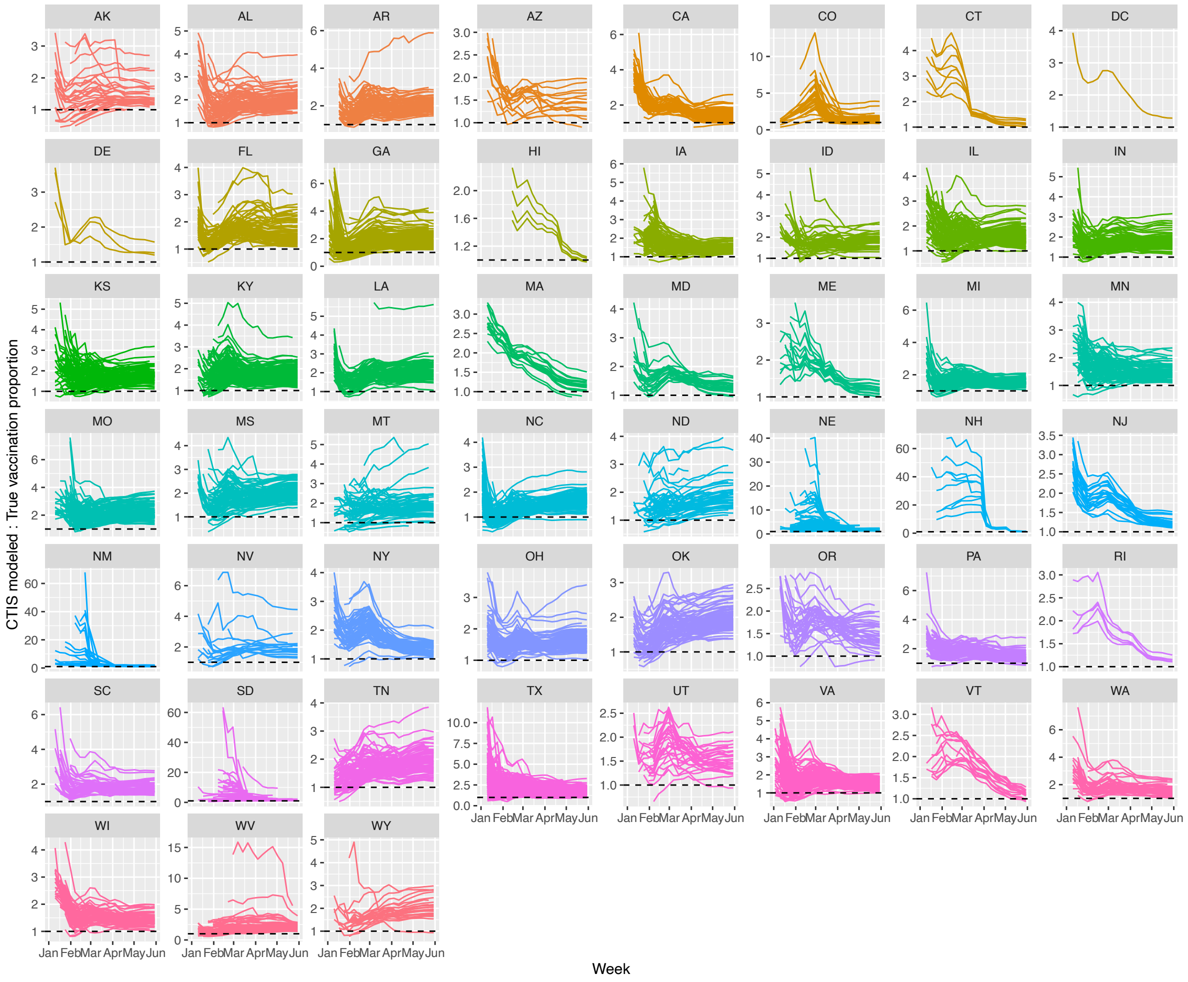

Figure S18. Motivation for bias correction using vaccination survey and ground-truth data. Ratio of modeled CTIS vaccination estimates to true vaccination proportions for each state over time (January 1 to June 30, 2021). Dashed line at a ratio of 1. CTIS survey estimates often substantially overestimate the fraction of vaccinated individuals.

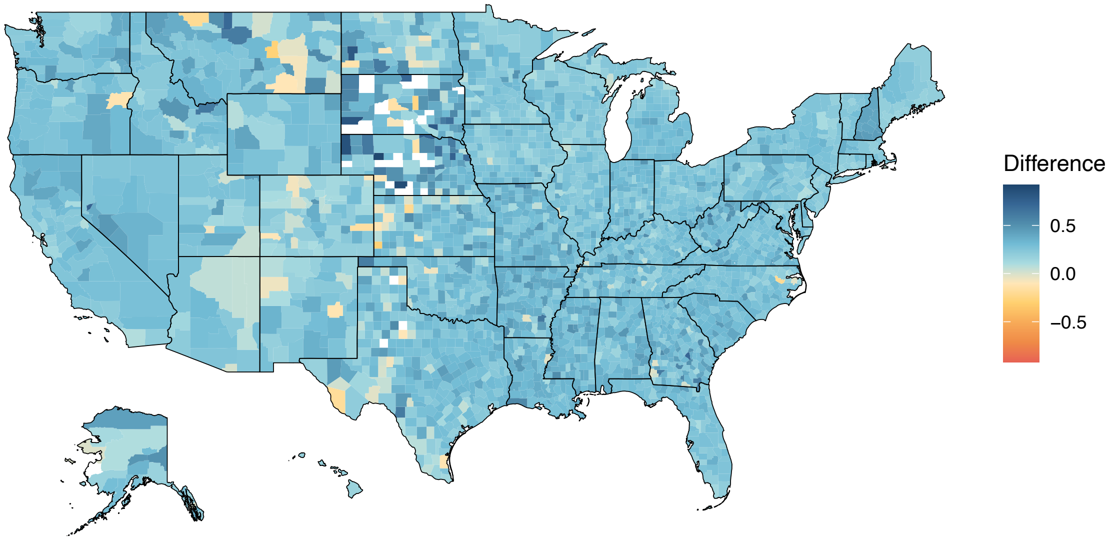

Figure S19. Map of differences between observed (raw) CTIS vaccination estimates and true COVID-19 vaccination coverage from the months of April and May. Note that these values are slightly different than the bias values used in our model which account for sample size issues in the CTIS vaccination estimates. Positive values indicate CTIS overestimated true vaccination coverage and negative values indicate underestimation of true coverage. There is some spatial heterogeneity in this bias.

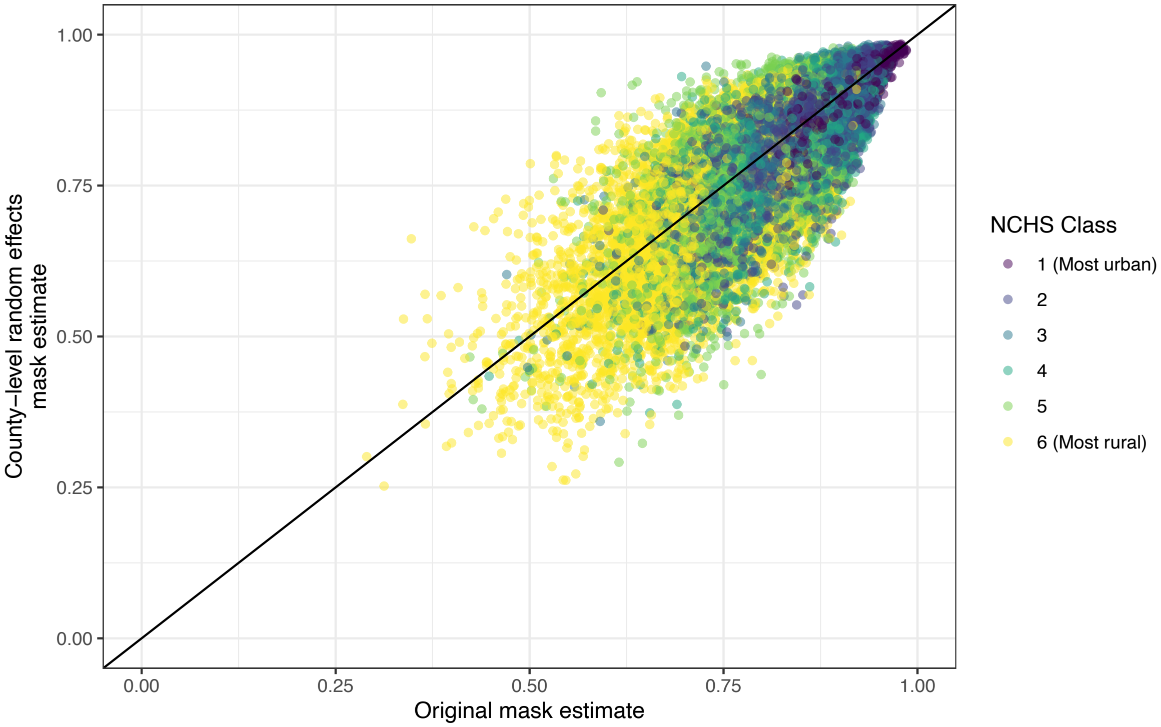

Figure S20. Estimates from model incorporating a county-level random effect (vertical axis) are, on average, similar to estimates from our original model without explicit spatial effects (horizontal axis). Both models use binomial regression on un-raked observations and do not incorporate the bias offset. Rural counties appear to show greater variability between the two models. Black line is y=x.

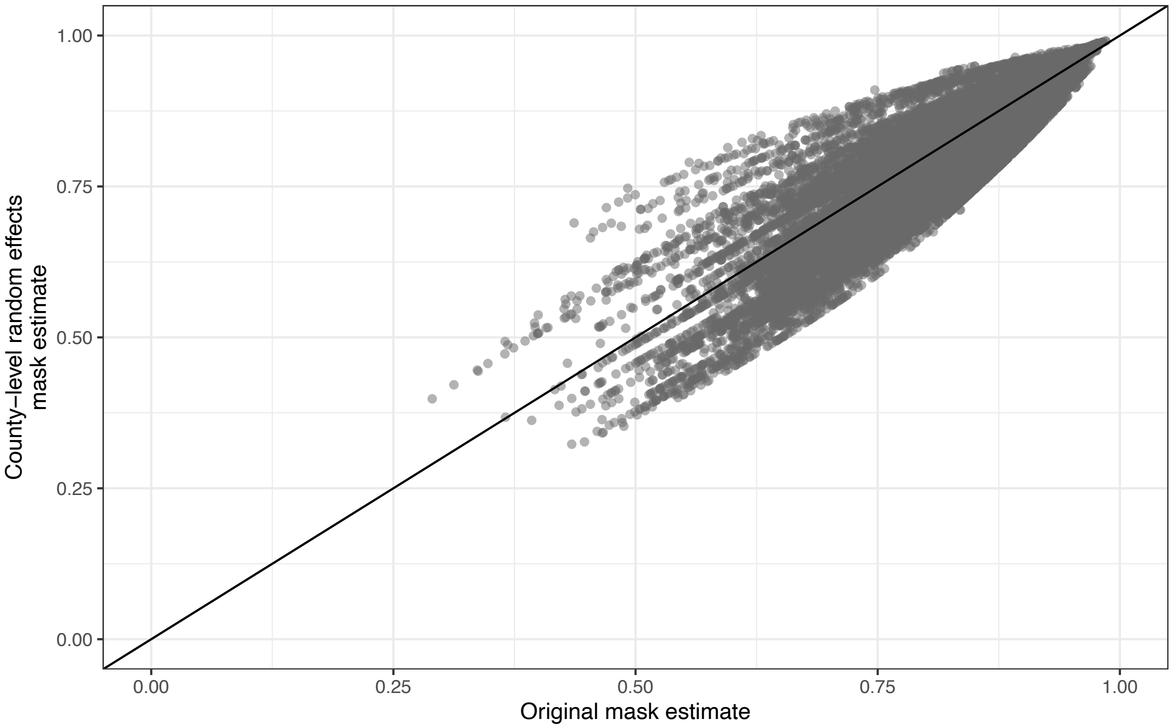

Figure S21. Estimates from model incorporating a state-level random effect (vertical axis) are, on average, similar to estimates from our original model without explicit spatial effects (horizontal axis). Both models use binomial regression on un-raked observations and do not incorporate the bias offset. The models with state-level random effects did not reliably converge and thus estimates should be interpreted with caution. Black line is y=x.

Month	Estimate	l-95% CI	u-95% CI
09/2020	0.51	0.51	0.52
10/2020	0.5	0.5	0.51
11/2020	0.49	0.48	0.5
12/2020	0.5	0.49	0.51
01/2021	0.53	0.52	0.54
02/2021	0.55	0.54	0.56
03/2021	0.55	0.54	0.55
04/2021	0.55	0.54	0.55
05/2021	0.45	0.45	0.46

Figure S22. Coefficients and 95% credible intervals for z-score(log_{10}(population density)) coefficient in the binomial regression model with raking and debiasing for each month. Coefficients are consistent over time.

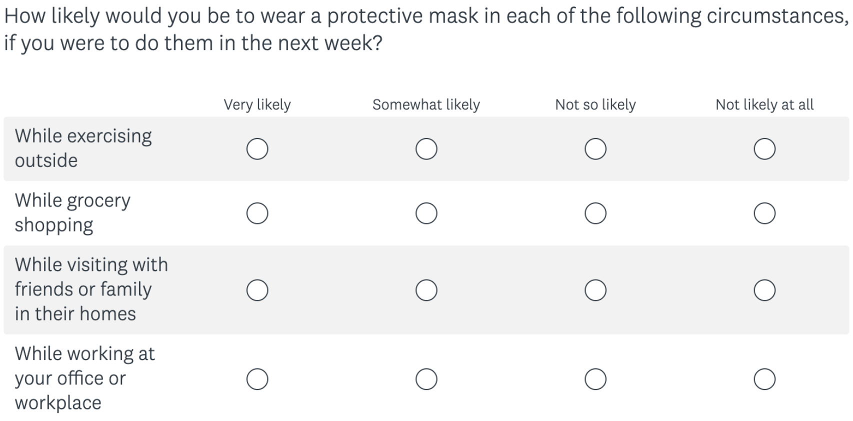

How likely would you be to wear a protective mask in each of the following circumstances, if you were to do them in the next week?

	Very likely	Somewhat likely	Not so likely	Not likely at all
While exercising outside	◯	◯	◯	◯
While grocery shopping	◯	◯	◯	◯
While visiting with friends or family in their homes	◯	◯	◯	◯
While working at your office or workplace	◯	◯	◯	◯

Figure S23. ONM question on masking behavior.

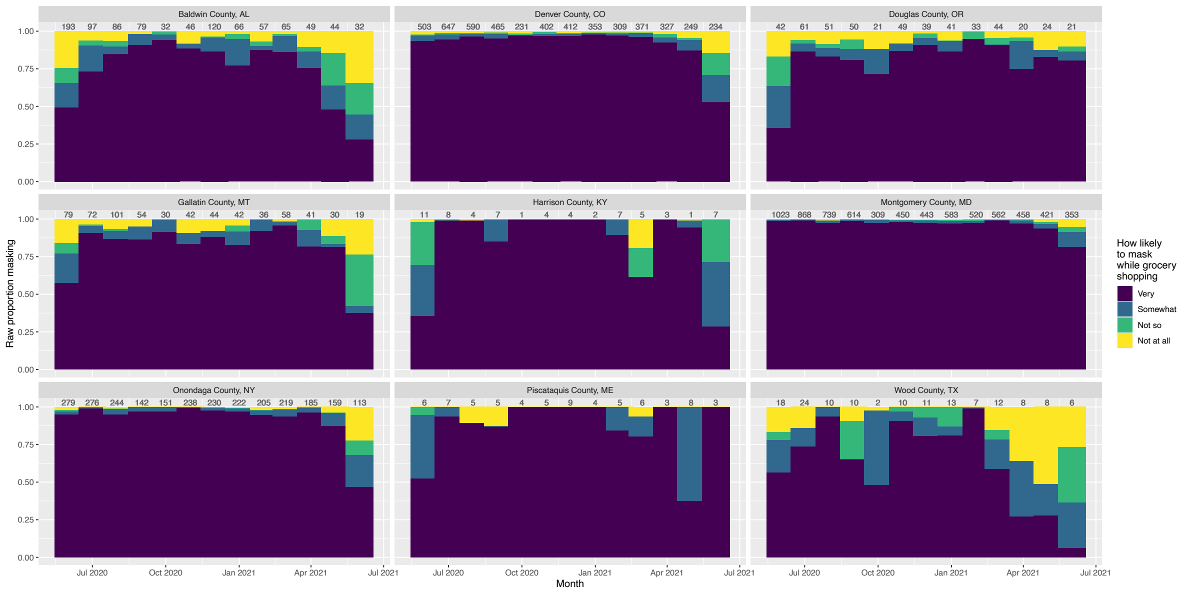

Figure S24. Raw proportions of each ONM response for a selection of counties spanning population densities across time. Number of responses in that county-month listed above each bar. NCHS urban-rural classifications are as follows: (1) Denver, CO; (2) Montgomery, MD; (3) Onondaga, NY; (4) Baldwin, AL; (5) Gallatin, MT; Douglas, OR; (6) Wood, TX; Piscataquis, ME; Harrison, KY.

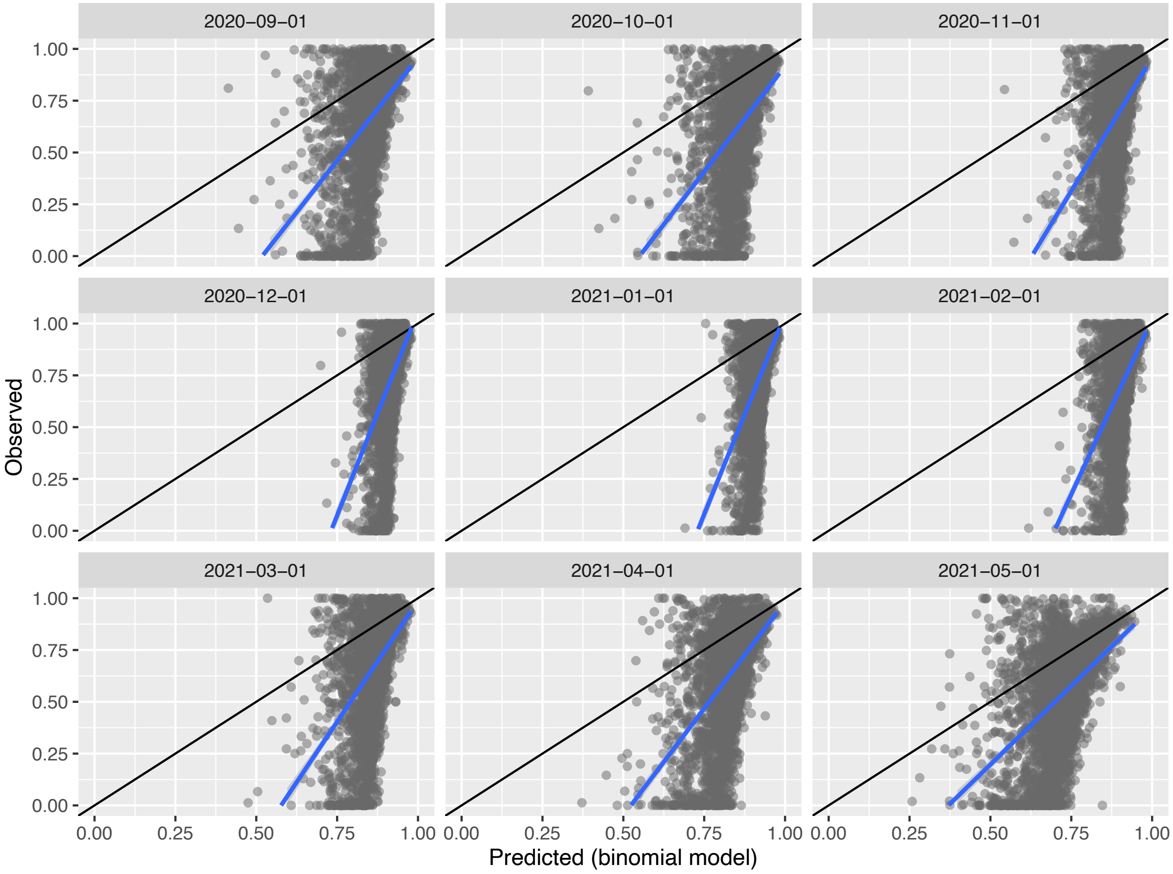

Figure S25. Observed versus predicted ONM masking estimates from binomial regression model. Black line shows y = x; blue line is a linear fit of the data. The linear fit is not close to the one-to-one line indicating poor model fit.

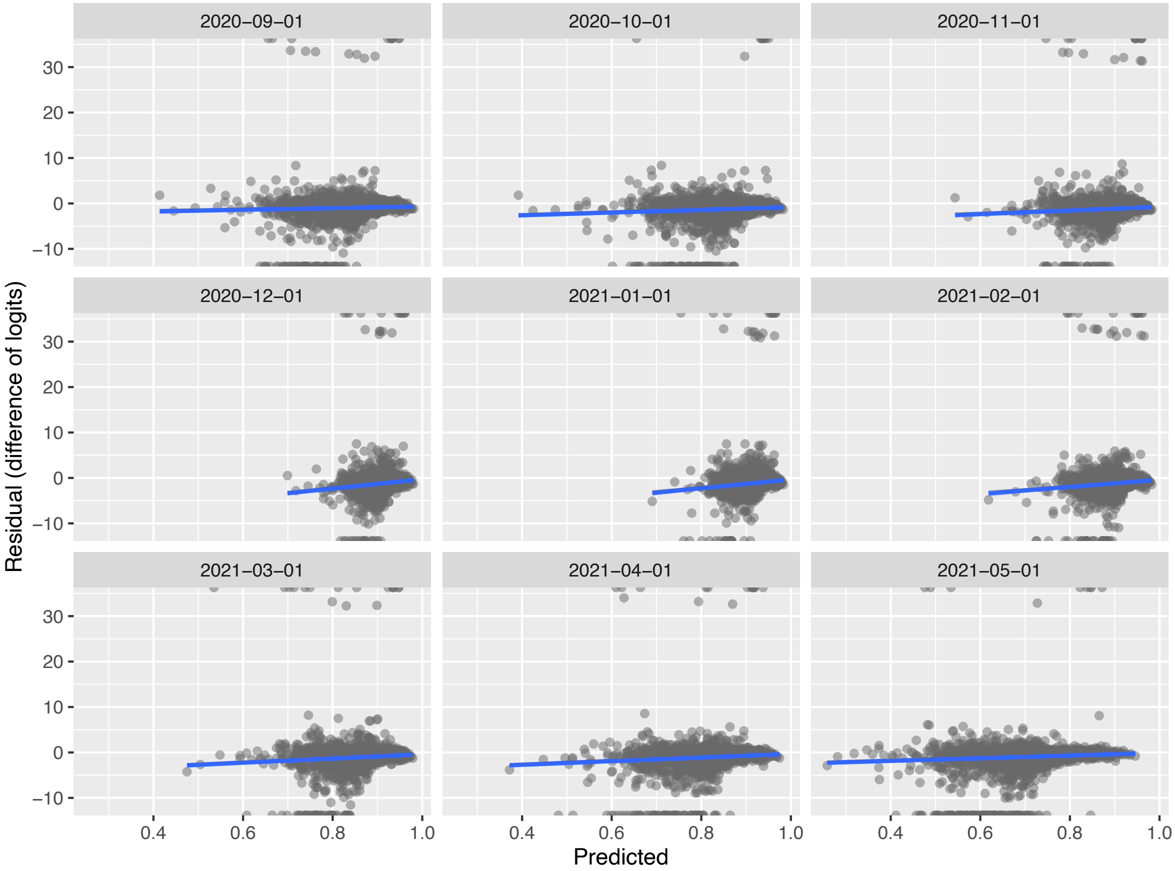

Figure S26. Observed versus residuals for ONM masking estimates. Blue line is a linear fit of the data. Residuals show weak association with predicted value, indicative of a mediocre model fit; there are also several outliers.

Table S1. Validation of model estimates from observational studies. Most studies that overlap with the survey period estimate the proportion of people wearing masks correctly within 10% of our debiased model estimates (green cells). Yellow cells denote literature estimates more than 10% higher than our estimates and red cells denote estimates more than 10% lower, with speculation on the reason for these differences provided in the similarly named column. Gray date cells indicate lack of overlap with our survey period.

Date (MM/YYYY)	Location	Lit. estimate	Model debiased estimate	Difference	Model fips	Model date (MM/YYYY)	Speculation on any differences	DOI/PMID/URL
09/2020	New Orleans, LA	0.32	0.78	-0.46	22071	09/2020	Observations of Bourbon Street which may be a sample disproportionately composed of tourists, people going out during a pandemic who may be less likely to wear a mask	10.1371/journal.pone.0261321
08/2020	Philadelphia, PA	0.43	0.85	-0.42	42101	09/2020	Time period different, mostly observed people outside at parks/streets where mask compliance may have been lower due to the outdoor nature	10.1016/j.pmedr.2021.101449
06/2021	Milwaukee, Waukesha, Ozaukee, Washington, and Sheboygan, WI	0.26	0.61	-0.35	55079, 55133, 55089, 55131, 55117	05/2021	Time period is different, averaging across several counties in Wisconsin	10.1101/2022.01.18.22269479
09/2020	Times Square, NY	0.65	0.91	-0.26	36061	09/2020	Sample may be disproportionately composed of tourists during a pandemic who may be less likely to wear a mask if choosing to travel during this time	10.1371/journal.pone.0261321
05/2020 - 06/2020	Wisconsin	0.41	0.54	-0.13	Average all WI	09/2020	Time period different, observed grocery stores across 20 counties, model estimate is averaged over the entirety of Wisconsin	10.1101/2020.06.09.20126946
03/2021 - 04/2021	Indianapolis, IN	0.74	0.85	-0.11	18097	03/2021 - 04/2021	Indoor basketball tournament (March Madness) may not be representative of typical people living in this area	10.1001/jama.2021.14057
06/2020 - 07/2020	Auburn-Opelika, AL	0.55	0.65	-0.10	1081	09/2020	Time period different, single estimate not recorded so averaging across Fig 2	10.1108/S0895-993520220000029006
10/2020	Philadelphia, PA	0.77	0.86	-0.09	42101	10/2020	Average across all observations in outside and retail sites	https://public.tableau.com/app/profile/city ofphiladelphia/viz/shared/MPS6SH482
06/2020 - 08/2020	Portland, OR and Toronto, CAN	0.68	0.77	-0.09	41051	09/2020	Time period different, higher levels of masking in Portland than Toronto but individual proportions not given	10.1136/bmjopen-2021-049389
09/2020	Philadelphia, PA	0.76	0.85	-0.09	42101	09/2020	Average across all observations in outside and retail sites	https://public.tableau.com/app/profile/city ofphiladelphia/viz/shared/MPS6SH482
12/2020	Philadelphia, PA	0.88	0.92	-0.04	42101	12/2020	Average across all observations in outside and retail sites	https://public.tableau.com/app/profile/city ofphiladelphia/viz/shared/MPS6SH482
11/2020	Philadelphia, PA	0.87	0.89	-0.02	42101	11/2020	Average across all observations in outside and retail sites	https://public.tableau.com/app/profile/city ofphiladelphia/viz/shared/MPS6SH482
09/2020	Little Italy, NYC, NY	0.9	0.91	-0.01	36061	09/2020		10.1371/journal.pone.0261321
12/2020	Louisville, KY	0.86	0.87	-0.01	21111	12/2020		10.1371/journal.pone.0248324
07/2020	Honolulu, HI	0.77	0.76	0.01	15003	09/2020	Time period different	PMID: 32914093
09/2020	Las Vegas, NV	0.86	0.85	0.01	32003	09/2020		10.1371/journal.pone.0261321
11/2020 - 05/2021	Marion County, IN	0.86	0.84	0.02	18097	11/2020 - 05/2021	Weekly data are available and are pretty consistent over time with a drop in last couple study weeks (May); anywhere between 80 - 90% usually	10.1097/PHH.0000000000001467
11/2020 - 05/2021	King County, WA	0.872	0.81	0.06	53033	11/2020 - 05/2021	Most observations in period from 12/2020 - 04/2021	10.1177/00333549221100795
05/2020	Chittenden County, VT	0.76	0.66	0.1	50007	09/2020	Time period different	10.1177/00333549211009496
09/2020	Key West, FL	0.66	0.56	0.1	12087	09/2020		10.1371/journal.pone.0261321
06/2020 - 07/2020	Greensboro, NC	< 0.75	0.72		37081	09/2020	Time period is different	10.23954/osj.v7i2.3084
04/2021 - 05/2021	Auburn-Opelika, AL	0.74	0.64	0.10	1081	04/2021 - 05/2021	Essential businesses, single estimate not recorded so averaging across Fig 3	10.1108/S0895-993520220000029006
08/2020	Milwaukee, Waukesha, Ozaukee, Washington, and Sheboygan, WI	0.96	0.7	0.26	55079, 55133, 55089, 55131, 55117	09/2020	Time period is different, averaging across several counties in Wisconsin	10.1371/journal.pone.0240785
09/2020 - 11/2020	South and West	0.77				10/2020	Location too vague for comparison, university campuses with mask mandates, 0.86 wore masks, but only 0.77 properly, 0.92 indoors properly	10.15585/mmwr.mm7006e1
