# Supplementary figures and images for: Spatiotemporal Trends in Self-Reported Mask-Wearing Behavior in the United States: Analysis of a Large Cross-sectional Survey
Source: JMIR Public Health Surveill. 2023 Mar 6;9:e42128. doi: 10.2196/42128 (PMC10028521; doi:10.2196/42128)

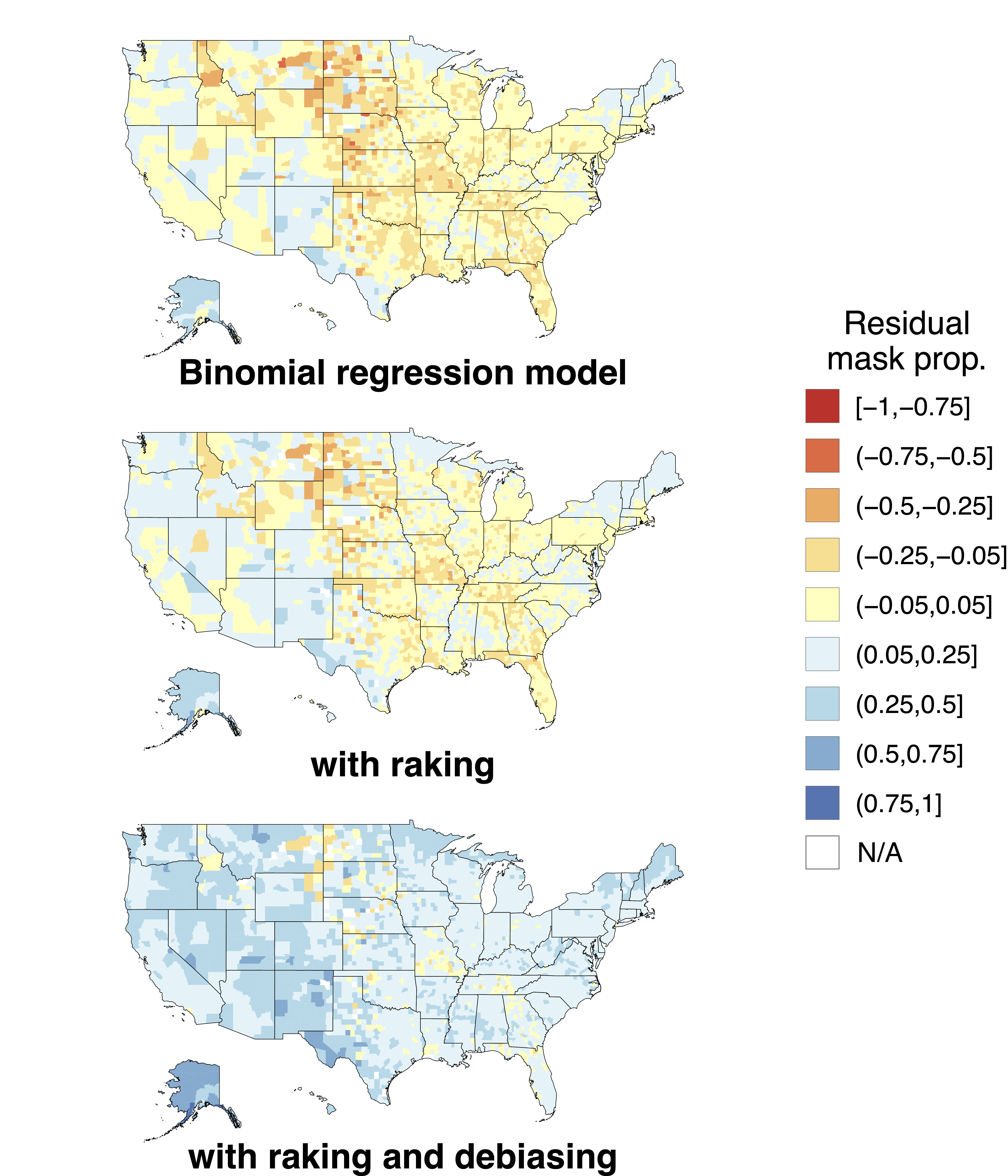

Supplement: Multimedia Appendix 2 [file publichealth_v9i1e42128_app2.png]

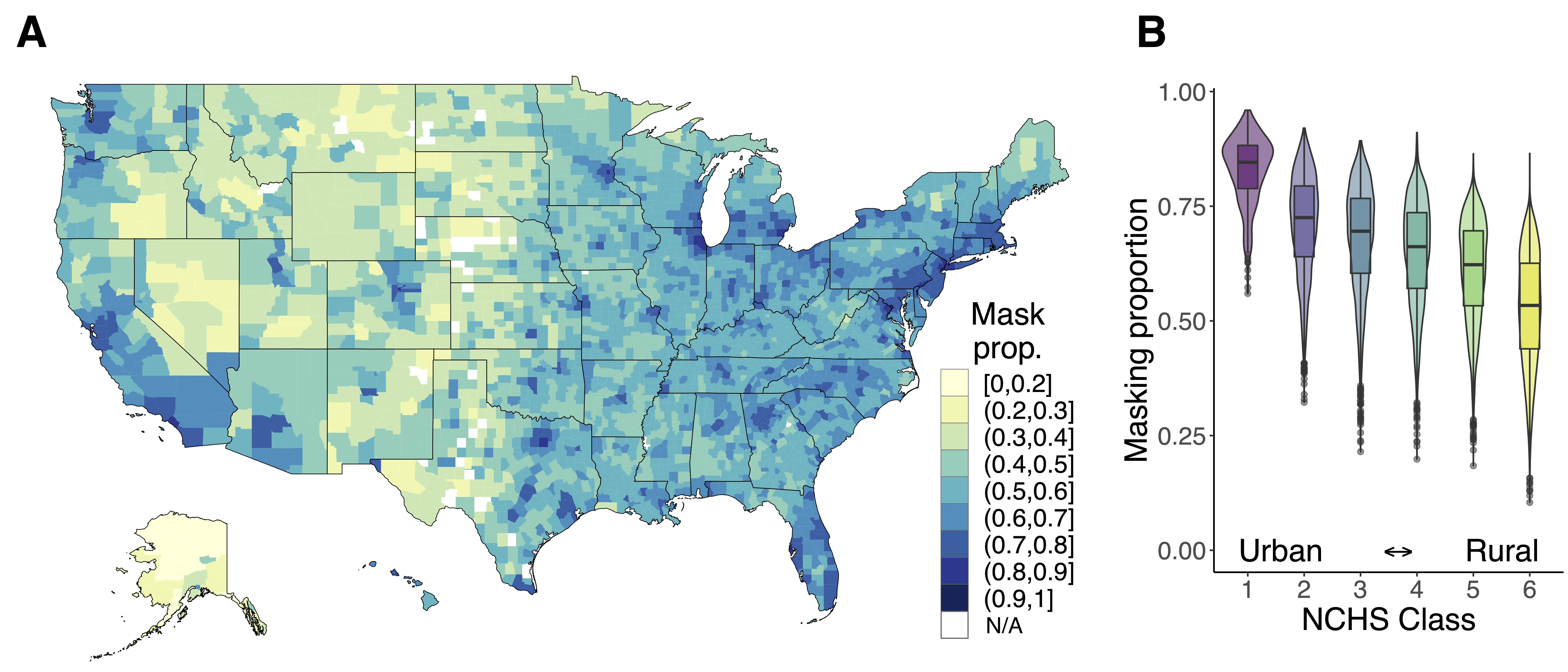

Supplement: Multimedia Appendix 3 [file publichealth_v9i1e42128_app3.png]

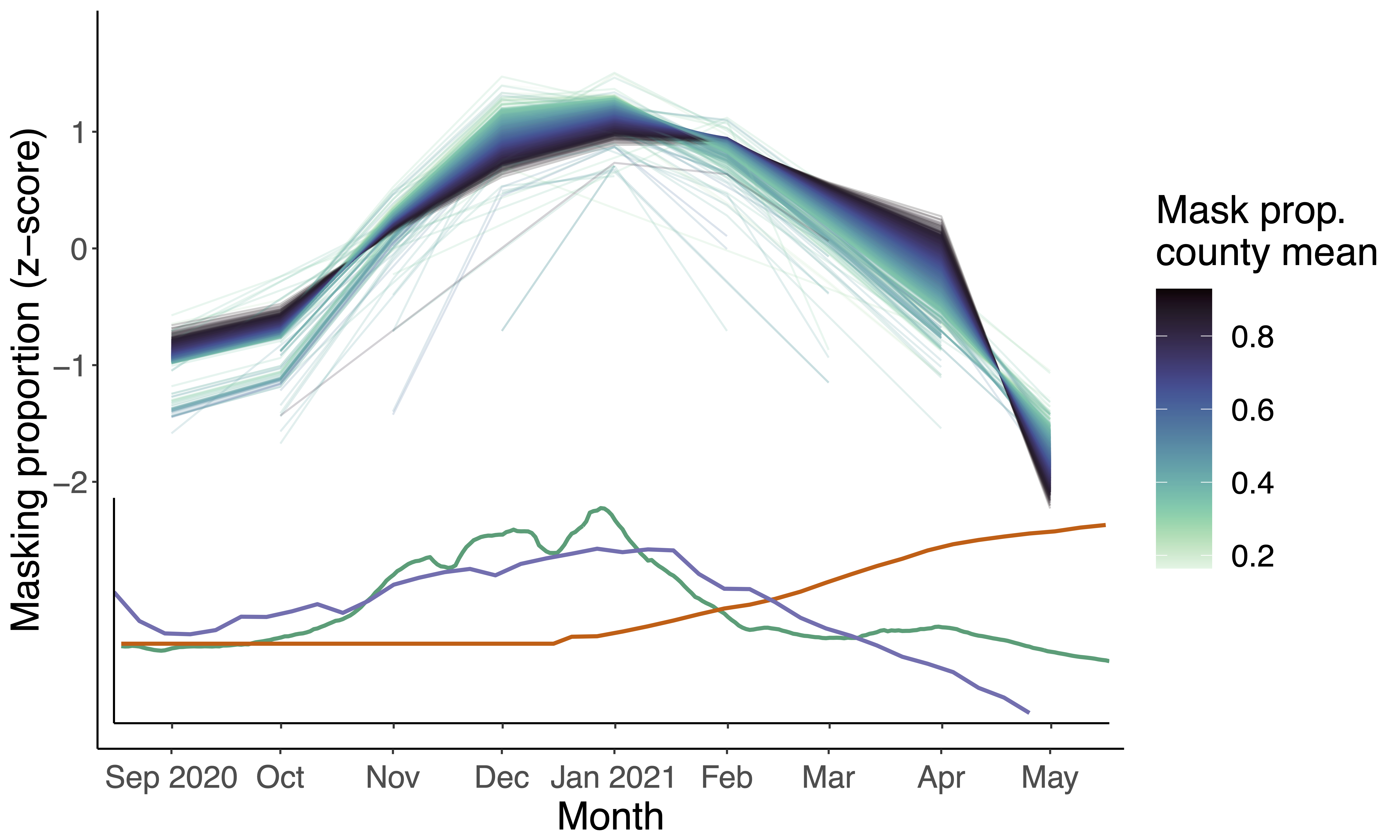

Supplement: Multimedia Appendix 4 [file publichealth_v9i1e42128_app4.png]

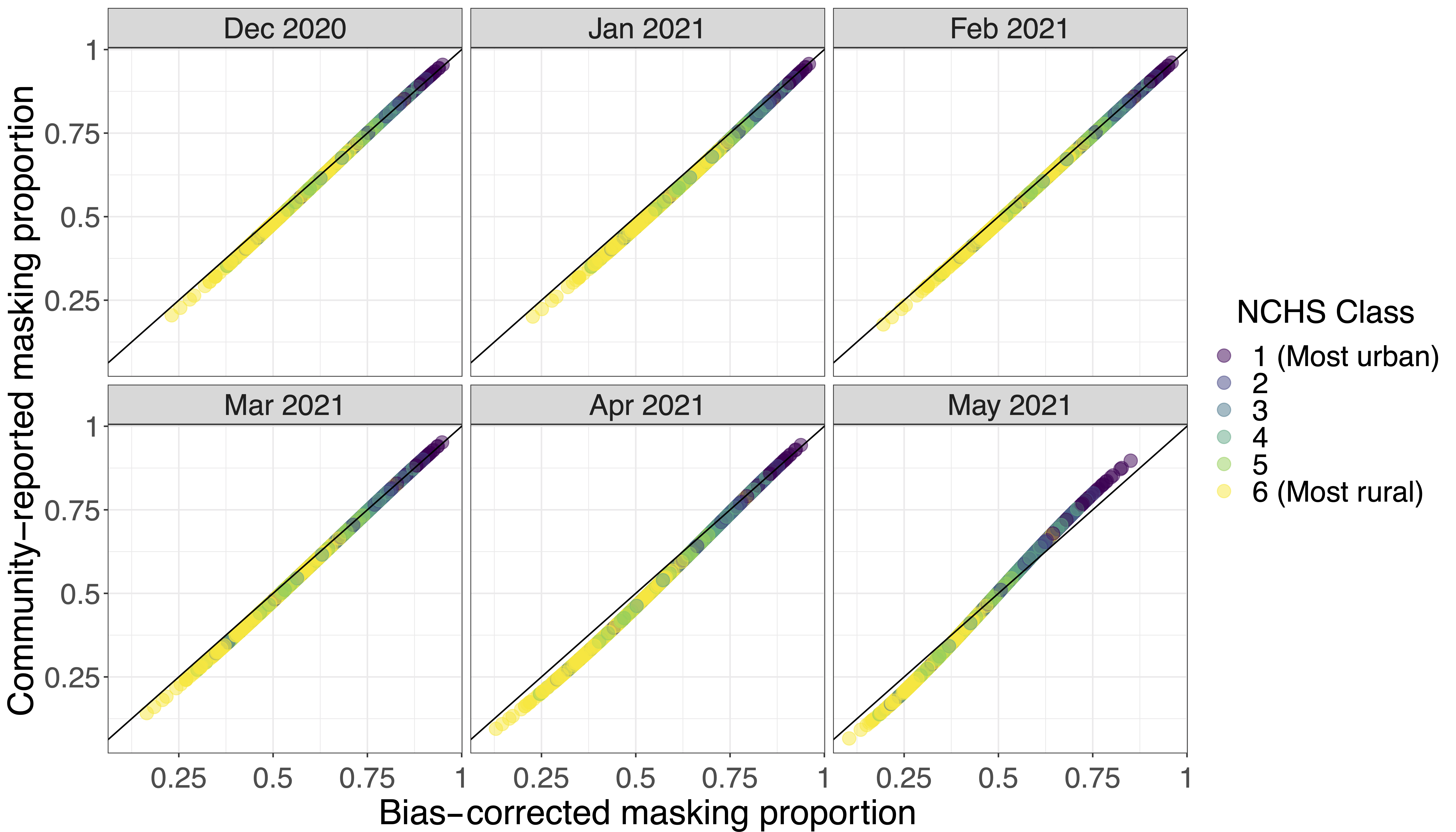

Supplement: Multimedia Appendix 5 [file publichealth_v9i1e42128_app5.png]
